# Interpretable machine learning models for predicting New-onset acute organ injury within 48 hours of admission in patients with community-acquired acute cholangitis

**DOI:** 10.3389/fmed.2026.1882266

**Published:** 2026-09-23

**Authors:** Zhi-Xiang Xu, Jun Yao, Kang-Pei Yan, Jian Xu

**Affiliations:** 1 Department of Gastroenterology, The Affiliated People's Hospital of Jiangsu University, Zhenjiang, Jiangsu, China; 2 Department of Geriatrics, The Affiliated People's Hospital of Jiangsu University, Zhenjiang, Jiangsu, China; 3 Department of Gastroenterology, The People's Hospital of Danyang, Affiliated Danyang Hospital of Nantong University, Zhenjiang, Jiangsu, China

**Keywords:** acute organ injury, community-acquired acute cholangitis, machine learning, predictive modeling, retrospective study

## Abstract

**Background:**

Community-acquired acute cholangitis (CAAC) can progress to acute organ injury (AOI), increasing poor prognosis risk. This study aimed to develop machine learning models using clinical information obtained before AOI onset to predict new-onset AOI within 48 h after admission in patients with CAAC.

**Methods:**

This single-center retrospective cohort study screened 487 patients with CAAC between January 2018 and December 2022; 456 without AOI at admission were included. The primary outcome was new-onset AOI within 48 h. Candidate predictors included demographic data, medical history, vital signs, laboratory parameters, and composite indices obtained after admission and before AOI onset. Patients were stratified into training and independent test sets at an 8:2 ratio. In the training set, univariable logistic regression (LR) was used for screening, followed by LR with least absolute shrinkage and selection operator (LASSO) penalty via 5-fold cross-validation. Five models were developed: random forest (RF), support vector machine (SVM), LR, neural network (NN), and adaptive boosting (AdaBoost). Optimization used 5-fold stratified cross-validation and grid search. The optimal model was selected by cross-validated area under the receiver operating characteristic curve (ROC-AUC) and evaluated for discrimination, calibration, and net clinical benefit. SHapley Additive exPlanations (SHAP) was used for interpretation.

**Results:**

RF and SVM achieved the highest mean cross-validated ROC-AUC (0.847 each). RF was selected per the prespecified tie-breaking protocol. In the independent test set, RF achieved an ROC-AUC of 0.820 [95% confidence interval (CI): 0.720–0.906], accuracy of 72.8%, sensitivity of 80.6%, specificity of 68.9%, positive predictive value (PPV) of 56.8%, negative predictive value (NPV) of 87.5%, and F1-score of 0.667. The Brier score was 0.156, calibration intercept 0.014, and calibration slope 1.252. Decision curve analysis (DCA) demonstrated a positive net clinical benefit across threshold probabilities ranging from 0.01 to 0.80. The eight key RF predictors were C-reactive protein (CRP), aspartate aminotransferase-to-platelet ratio index (APRI), MAP, prognostic nutritional index (PNI), gamma-glutamyl transferase-to-albumin ratio (GAR), age, neutrophil-to-lymphocyte ratio (NLR), and drink history.

**Conclusion:**

The RF model predicted new-onset AOI within 48 h in patients with CAAC and provided interpretable individual risk information through SHAP analysis.

## 1 Introduction

Acute cholangitis (AC) is an acute inflammatory condition primarily caused by biliary obstruction, leading to bile stasis and infection (1, 2). Common etiologies include choledocholithiasis, benign or malignant strictures, and pancreatitis (3, 4). Without prompt intervention, AC can progress to sepsis and AOI, significantly increasing mortality (5, 6). CAAC, distinct from hospital-onset cases, presents with unique epidemiological and microbiological features, posing a substantial threat to patient outcomes and public health (7).

AOI, characterized by rapid dysfunction of organs such as the kidneys, liver, and lungs, is a critical complication of CAAC. Its development drastically elevates treatment complexity, healthcare costs, and mortality rates. Notably, not all patients who develop AOI have evident organ injury at the time of admission. Some patients with CAAC do not meet the diagnostic criteria for AOI upon admission but may rapidly develop new-onset AOI during the early stage after admission as biliary infection persists, systemic inflammatory responses intensify, circulatory perfusion deteriorates, and coagulation and metabolic disturbances progress. Therefore, compared with identifying organ injury that is already present at admission, predicting the risk of AOI within the subsequent 48 h among patients who have not yet developed AOI, using clinical information obtained early after admission and before AOI onset, is more consistent with the clinical need for early warning. Early identification of high-risk patients is, therefore, paramount for initiating timely interventions like biliary drainage, which can improve prognosis (8).

Traditional risk prediction models, like LR, have been utilized but possess inherent limitations (9). They often struggle with subjective variable selection, complex non-linear relationships, and imbalanced or missing data—common challenges in clinical datasets.

As a data-driven paradigm, machine learning is particularly adept at capturing complex nonlinear relationships in high-dimensional data, offering substantial promise for enhancing predictive performance in clinical settings. Although machine learning has been increasingly adopted in prognostic research (10, 11), evidence remains scarce concerning the use of pre-AOI-onset clinical information to predict new-onset organ injury within the first 48 h of admission, especially in patients with CAAC without baseline organ involvement.

This study focused on patients with CAAC without AOI at hospital admission and used demographic characteristics, clinical variables, and laboratory measurements obtained after admission but before AOI onset to develop and evaluate multiple machine-learning prediction models for identifying patients at high risk of developing new-onset AOI within the subsequent 48 h. SHAP was further applied to interpret the best-performing model and quantify the contribution of major predictors to model output, with the aim of supporting early risk stratification, intensified monitoring, and individualized clinical management in patients with CAAC.

## 2 Methods

### 2.1 Study design and population

This single-center retrospective cohort study was approved by the Ethics Committee of the Affiliated People's Hospital of Jiangsu University (Approval No. KYW001-13), with a waiver of informed consent. A total of 487 patients initially diagnosed with CAAC between January 2018 and December 2022 were screened. After application of the predefined exclusion criteria, 456 patients were included in the final analysis, as shown in Figure 1.

**Figure 1:**
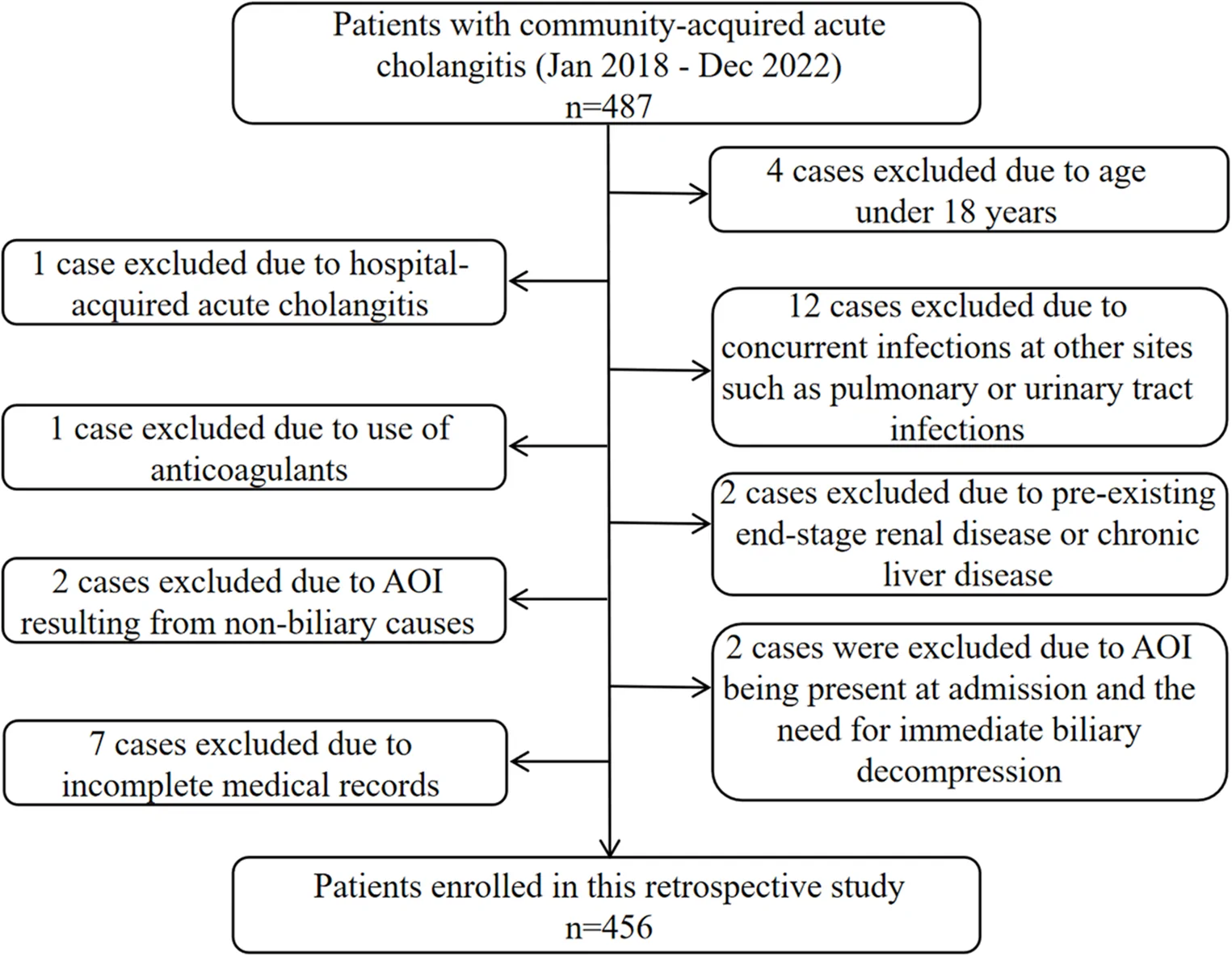
Participant selection flowchart.

The exclusion criteria were as follows: hospital-acquired acute cholangitis; age <18 years; concurrent infections at other sites, such as pulmonary or urinary tract infections; use of anticoagulants (e.g., warfarin); pre-existing end-stage renal disease or chronic liver disease; AOI attributable to non-biliary causes (e.g., recent major surgery or trauma); AOI already present at admission, generally requiring immediate biliary decompression; and incomplete medical records.

Hospital admission was defined as the study baseline. The target population comprised patients with CAAC who had not developed AOI at baseline. Accordingly, patients who already met the diagnostic criteria for AOI at admission were excluded because the target outcome was already present and these patients generally required urgent biliary decompression under the predefined clinical management pathway at our institution. The primary outcome was defined as the first occurrence of incident AOI within 48 h after admission. Thus, the study was designed to predict early progression from CAAC without AOI at baseline to incident AOI during hospitalization, rather than to identify pre-existing organ injury at admission.

### 2.2 Data collection and variable definitions

Baseline clinical data were defined using a fixed time window anchored to hospital admission. For each candidate predictor, the first available measurement recorded within 12 h after admission was used for analysis. Measurements obtained after the onset of AOI were not used for predictor derivation. Candidate predictors were categorized into the following three domains: (1) Demographics and Medical History, including age, sex, smoking history, alcohol use, and history of hypertension, diabetes, cardiovascular disease, and gallstone disease; (2) Vital signs and Physical Examination Parameters, including body temperature, heart rate, mean arterial pressure (MAP), and body mass index (BMI). MAP was calculated as diastolic blood pressure + 1/3  ×  (systolic blood pressure−diastolic blood pressure), using the first blood pressure measurement obtained after admission and before fluid resuscitation or initiation of vasoactive therapy. BMI was calculated as body weight (kg) divided by height squared (m^2^); (3) Laboratory Parameters, including complete blood count parameters (CRP, neutrophil count, lymphocyte count, hemoglobin, and platelet count), and biochemical profiles (globulin, albumin, aspartate aminotransferase [AST], alanine aminotransferase [ALT], gamma-glutamyl transferase [GGT], alkaline phosphatase [ALP], triglyceride, and cholesterol).

Furthermore, twelve immunoinflammatory and nutritional biomarkers were derived from the original data. These included the albumin-to-alkaline phosphatase ratio (AAPR) = albumin (g/L)/ALP (U/L); albumin-to-globulin ratio (AGR) = albumin (g/L)/globulin (g/L); GAR = GGT (U/L)/albumin (g/L); NLR = neutrophil count ( × 10⁹/L)/lymphocyte count (×10⁹/L); platelet-to-lymphocyte ratio (PLR) = platelet count (×10⁹/L)/lymphocyte count (×10⁹/L); PNI = albumin (g/L) + 5 × lymphocyte count (×10⁹/L); systemic immune-inflammation index (SII) = platelet count(×10⁹/L) × neutrophil count(×10⁹/L)/lymphocyte count(×10⁹/L); lymphocyte-to-monocyte ratio (LMR) = lymphocyte count (×10⁹/L)/monocyte count (×10⁹/L); APRI = AST (U/L)/platelet count (×10⁹/L) × 100; aspartate aminotransferase-to-neutrophil ratio index (ANRI) = AST (U/L)/neutrophil count (×10⁹/L); aspartate aminotransferase-to-lymphocyte ratio index (ALRI) = AST (U/L)/lymphocyte count (×10⁹/L); and the Controlling Nutritional Status (CONUT) score, which was calculated based on serum albumin, lymphocyte count, and cholesterol level, with patients subsequently stratified into low and high groups using the median score as the cutoff (12, 13).

### 2.3 Outcome variable

The outcome variable, AOI, is a composite endpoint encompassing six major organ systems. Diagnoses are based on internationally recognized criteria: acute kidney injury is defined according to the Kidney Disease: Improving Global Outcomes (KDIGO) guidelines, based on an increase in serum creatinine or reduced urine output (14); acute lung injury is defined as an arterial oxygen partial pressure to fractional inspired oxygen ratio (PaO₂/FiO₂) ≤ 300 mm Hg with bilateral infiltrates and no evidence of heart failure (15); acute liver injury is defined as a serum bilirubin level >2 mg/dL combined with an INR >1.5 (16, 17); acute neurological injury is defined as a newly developed decrease of ≥2 points in the Glasgow Coma Scale score from the admission baseline, with a new acute intracranial lesion confirmed by cranial CT and/or MRI (18). Given the retrospective nature of the study, neuroimaging evidence was additionally required to improve the objectivity and reproducibility of neurological outcome ascertainment and to reduce potential misclassification caused by non-structural factors affecting consciousness. This definition therefore prioritized specificity and may not capture septic encephalopathy without structural intracranial lesions. Acute myocardial injury is defined as a ≥ 20% change in consecutive cardiac troponin I measurements (19, 20); and acute circulatory injury is defined by the need for vasopressors to maintain adequate blood pressure (21, 22).

Starting from the time of hospital admission, the observation period was calculated. Patients who first met the diagnostic criteria for AOI in any of the aforementioned organ systems within 48 h after admission were defined as having a positive primary outcome. Patients who did not develop AOI within 48 h after admission were defined as having a negative primary outcome. AOI events that first occurred more than 48 h after admission were not included in the primary 48-hour predictive endpoint of this study.

### 2.4 Dataset partitioning and preprocessing

Before outcome-related variable selection, the complete cohort was stratified and randomly divided into a training set and an independent test set at an 8:2 ratio according to the 48-hour AOI outcome to maintain comparable event distributions. The training set was used for variable screening, preprocessing rule establishment, model training, hyperparameter optimization, and decision threshold determination, whereas the independent test set was reserved exclusively for final model evaluation. All preprocessing procedures were established using the training set only to avoid information leakage.

Categorical variables were predefined according to clinical characteristics, including sex, smoking status, drink status, diabetes, hypertension, cardiovascular disease, cholelithiasis, and CONUT categories. Other numerical variables were treated as continuous variables. Missing-value handling procedures were fitted exclusively within the training set. Continuous variables were assessed using the Kolmogorov–Smirnov test; normally distributed variables were imputed using the mean, whereas non-normally distributed variables were imputed using the median. Categorical variables were imputed using the mode. The derived imputation rules were subsequently applied unchanged to the independent test set.

For machine learning modeling, categorical variables were transformed using one-hot encoding. Continuous variables were standardized using StandardScaler, with all transformation parameters estimated from the training set.

### 2.5 Candidate variable screening, LASSO feature selection, and multicollinearity assessment

Univariable LR analyses were performed exclusively in the training set. For continuous variables, odds ratio (OR), 95% CI, and *p* values were calculated per unit increase. For categorical variables, the first category was used as the reference level, and global *p* values were obtained using likelihood ratio testing.

Variables with *p* < 0.01 were considered candidates according to the prespecified analysis plan. Continuous variables were selected according to regression *p* values, whereas categorical variables were selected according to global *p* values.

Candidate variables were subsequently entered into logistic-LASSO regression. L1-penalized LR was applied, with lambda defined as 1/C. A total of 120 candidate lambda values ranging from 10⁻⁴ to 10^3^ were evaluated using 5-fold stratified cross-validation. The lambda_min and lambda_1se values were obtained based on mean binomial deviance and its standard error. To obtain a stable and parsimonious feature set, lambda_1se was selected, and variables with absolute coefficients >1 × 10⁻⁶ were retained.

Pearson correlation matrices and variance inflation factor (VIF) were calculated for the selected features in the training set to evaluate potential multicollinearity. These analyses were performed for descriptive and diagnostic purposes only and were not used for automatic feature elimination.

### 2.6 Machine learning model development and optimization

Five classification models were developed using LASSO-selected features: RF, SVM, LR, NN, and AdaBoost. All models were optimized using grid search combined with 5-fold stratified cross-validation in the training set, with ROC-AUC as the optimization metric. The predefined hyperparameter search spaces included the number of estimators, maximum tree depth, minimum samples required for splitting, and maximum features for RF; C, gamma, and kernel type for SVM; C and l1_ratio for LR; hidden-layer size, alpha, learning rate, and batch size for NN; and the number of estimators and learning rate for AdaBoost. The detailed hyperparameter search spaces are provided in Supplementary Table 1. The model achieving the highest mean cross-validated ROC-AUC was defined as the preferred model. In the event of a tie among multiple models, the final optimal model was determined according to a prespecified selection protocol, prioritizing models with better clinical interpretability. Out-of-fold (OOF) predicted probabilities were then generated for the optimal model using the same 5-fold stratified cross-validation procedure for internal discriminative assessment and threshold determination.

### 2.7 Model performance evaluation and calibration analysis

The primary decision threshold was determined exclusively from training-set OOF predictions to avoid any optimization bias from the independent test set. The Youden index was calculated based on the OOF-derived receiver operating characteristic (ROC) curve using the formula: Youden index = sensitivity + specificity−1. The threshold corresponding to the maximum Youden index was selected as the optimal cutoff and subsequently fixed for all evaluations in the independent test cohort. Model performance was assessed in the independent test set, with the ROC-AUC considered the primary performance metric. The 95% CI of ROC-AUC was estimated using 1,000 bootstrap resampling iterations. Using the predefined threshold, classification performance was further evaluated by calculating accuracy, sensitivity, specificity, PPV, NPV, F1-score, and confusion matrix components. In addition, precision-recall curves and average precision (AP) were assessed to further characterize model discrimination, particularly in the context of potential class imbalance. Threshold sensitivity analysis was performed by evaluating alternative decision thresholds ranging from 0.05 to 0.95 with an increment of 0.05, together with the OOF-derived Youden threshold, to examine the robustness of model performance across different cutoff values. Model calibration was evaluated using calibration curves constructed according to deciles of predicted probabilities. The Brier score, Hosmer–Lemeshow goodness-of-fit test, calibration intercept, and calibration slope were calculated to quantify the agreement between predicted probabilities and observed outcomes.

### 2.8 Clinical utility analysis and model interpretation

DCA was performed for the optimal model in the independent test cohort to evaluate its potential clinical utility across a range of decision thresholds. The threshold probability was set from 0.01 to 0.80 with an interval of 0.001, and the net benefit was calculated at each threshold probability. The performance of the model was compared with the “treat-all” and “treat-none” strategies. The 95% CI of the net benefit were estimated using 1,000 bootstrap resampling iterations.

The interpretability of the optimal machine learning model was further assessed using SHAP analysis. For tree-based models, SHAP values were calculated using the TreeExplainer algorithm. A maximum of 80 patients were randomly selected from the independent test cohort for global interpretation analysis. First, a SHAP feature importance bar plot was generated based on the mean absolute SHAP values of each feature across all selected samples, and features were ranked according to their overall contribution to the model output. A higher mean absolute SHAP value indicated a greater overall contribution of the corresponding feature to model prediction; however, this metric reflects the magnitude of influence on model output rather than a causal relationship.

Subsequently, SHAP beeswarm plots were generated to visualize the direction, magnitude, and distribution of feature contributions across individual samples. Each point in the plot represents the SHAP value of a specific feature for an individual sample. The *x*-axis represents the SHAP value, indicating both the direction and magnitude of the feature contribution to model prediction. A positive SHAP value indicates that the feature drives the model output toward the positive outcome (i.e., AOI diagnosis), whereas a negative SHAP value indicates the opposite effect. The color of each point represents the original feature value, with red indicating higher feature values and blue indicating lower feature values. Therefore, the beeswarm plot not only illustrates the relative importance of each feature but also demonstrates whether higher or lower feature values tend to increase or decrease the predicted probability of AOI.

Individual-level interpretation was further performed using SHAP force plots. Based on the Youden index-derived optimal threshold determined from OOF predictions in the training cohort, representative cases were selected from the independent test cohort, including the first true-positive case with confirmed AOI occurrence within 48 h and a predicted probability above the threshold, as well as the first true-negative case without AOI occurrence and a predicted probability below the threshold. SHAP force plots were used to illustrate how individual predictions were derived from the combined contributions of multiple features. Starting from the baseline value (i.e., the average model output across the dataset), each feature contributed positively or negatively through its corresponding Shapley value, ultimately resulting in the final predicted probability for each individual. Positive Shapley values indicated that the corresponding feature shifted the prediction toward AOI diagnosis, whereas negative Shapley values indicated a contribution in the opposite direction. This individualized interpretation enabled visualization of the key factors driving prediction outcomes at the patient level and improved the interpretability of the machine learning model.

### 2.9 Statistical software

All data processing, statistical analyses, and machine learning modeling were performed using Python version 3.13.7. The main packages included NumPy (2.4.6), pandas (3.0.3), statsmodels (0.14.6), SciPy (1.18.0), scikit-learn (1.9.0), SHAP (0.52.0), Matplotlib (3.11.0), and Seaborn (0.13.2).

## 3 Results

### 3.1 Study population and dataset partitioning

A total of 487 patients with CAAC were initially screened, of whom 456 met the predefined inclusion criteria and had no AOI at admission and were included in the final analysis. During hospitalization, 155 patients (34.0%) developed new-onset AOI within 48 h, whereas the remaining 301 patients (66.0%) did not experience AOI. Among patients with AOI, acute kidney injury was the most common type (n = 83), followed by acute lung injury (*n* = 32), acute liver injury (*n* = 21), acute myocardial injury (*n* = 12), acute circulatory injury (*n* = 5), and acute neurological injury (*n* = 2).

Using a predefined stratified random sampling strategy with an 8:2 ratio, the cohort was randomly divided into a training set (*n* = 364) and an independent test set (*n* = 92). During data preprocessing, only one patient in the training set had missing information regarding a history of gallstone disease, and this missing value was imputed based on the distribution of the training set data. No missing data were present in the independent test set; therefore, the original data distribution was retained without imputation to avoid potential data leakage. In the independent test set, 31 patients (33.7%) developed AOI within 48 h, whereas 61 patients (66.3%) remained free of AOI. The incidence of 48-hour AOI was comparable between the training and test sets (34.0% vs. 33.7%), indicating a balanced distribution of the outcome between the two cohorts.

### 3.2 Univariate screening, LASSO regression, and multicollinearity assessment

In the training cohort, univariate LR identified 11 candidate variables with *p* < 0.01, including Age, MAP, CRP, GAR, NLR, PNI, LMR, APRI, ALRI, Drink, and CONUT (Table 1). These variables were subsequently entered into logistic LASSO regression analysis. Five-fold cross-validation identified a lambda_min value of 1.50131 and a lambda_1se value of 7.62699. According to the predefined feature selection strategy, the lambda_1se criterion was applied, resulting in the retention of eight variables with non-zero coefficients: CRP, APRI, MAP, PNI, GAR, Age, NLR, and Drink. The LASSO coefficient profiles and five-fold cross-validation results are shown in Figures 2, 3.

**Table 1:** Univariate logistic regression analysis of candidate predictors associated with 48-hour new-onset AOI in the training set (*n* = 364).

Variable	OR (95% CI)	*p* value	Global *p* value
Age	1.030 (1.013–1.047)	<0.001	<0.001
Body temperature	0.985 (0.928–1.045)	0.613	0.613
Heart rate	1.007 (0.993–1.022)	0.316	0.316
BMI	0.930 (0.869–0.994)	0.033	0.033
MAP	0.935 (0.915–0.955)	<0.001	<0.001
CRP	1.013 (1.009–1.017)	<0.001	<0.001
Hemoglobin	0.988 (0.977–1.000)	0.042	0.042
AAPR	3.231 (0.699–14.926)	0.133	0.133
AGR	0.907 (0.451–1.826)	0.785	0.785
GAR	0.949 (0.923–0.976)	<0.001	<0.001
NLR	1.032 (1.018–1.046)	<0.001	<0.001
PLR	0.999 (0.998–1.000)	0.241	0.241
PNI	0.885 (0.847–0.924)	<0.001	<0.001
SII	1.000 (1.000–1.000)	0.628	0.628
LMR	0.742 (0.618–0.890)	0.001	0.001
APRI	1.475 (1.269–1.714)	<0.001	<0.001
ANRI	1.003 (0.999–1.006)	0.134	0.134
ALRI	1.001 (1.000–1.001)	<0.001	<0.001
Triglyceride	1.023 (0.751–1.394)	0.886	0.886
Sex	0.912 (0.589–1.413)	0.681	0.681
Smoke	0.789 (0.461–1.349)	0.386	0.381
Drink	2.029 (1.283–3.208)	0.002	0.003
Diabetes	1.849 (1.087–3.144)	0.023	0.024
Hypertension	1.222 (0.792–1.887)	0.365	0.365
Cardiacdisease	1.975 (1.080–3.612)	0.027	0.028
Gallstones	1.124 (0.654–1.934)	0.672	0.671
CONUT	2.739 (1.741–4.310)	<0.001	<0.001

AOI, acute organ injury; OR, odds ratio; CI, confidence interval; BMI, body mass index; MAP, mean arterial pressure; CRP, C-reactive protein; AAPR, albumin-to-alkaline phosphatase ratio; AGR, albumin-to-globulin ratio; GAR, gamma-glutamyl transferase-to-albumin ratio; NLR, neutrophil-to-lymphocyte ratio; PLR, platelet-to-lymphocyte ratio; PNI, prognostic nutritional index; SII, systemic immune-inflammation index; LMR, lymphocyte-to-monocyte ratio; APRI, aspartate aminotransferase-to-platelet ratio index; ANRI, aspartate aminotransferase-to-neutrophil ratio index; ALRI, aspartate aminotransferase-to-lymphocyte ratio index; CONUT, controlling nutritional status score.

**Figure 2:**
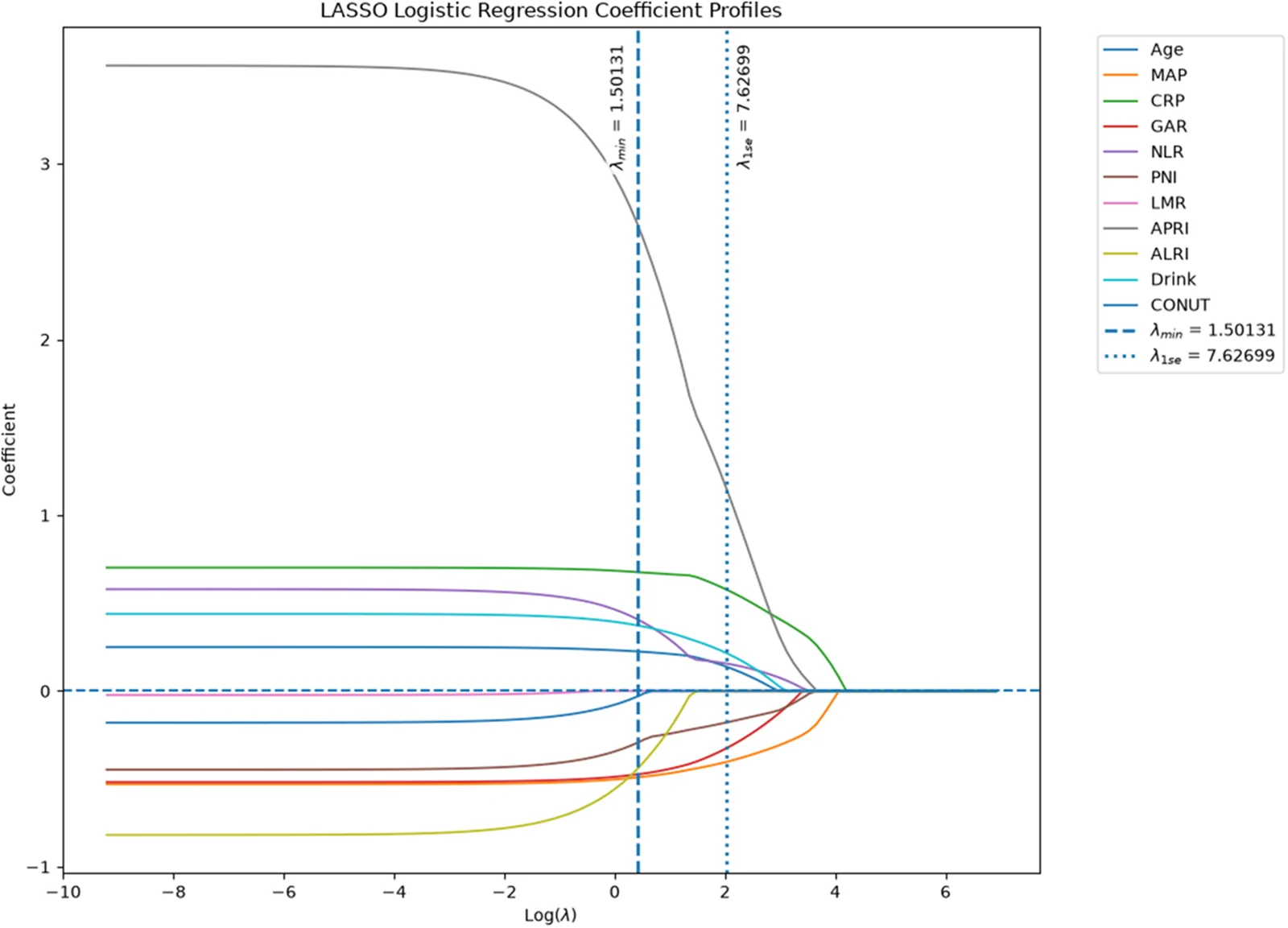
LASSO coefficient paths for predictor selection. The *x*-axis shows the log-transformed penalty parameter log(*λ*), and the *y*-axis shows the regression coefficients of the standardized candidate predictors, with each colored line representing one predictor. The vertical dashed line marks *λ*_mi*n* = 1.50131, at which the cross-validated binomial deviance is minimized; the vertical dotted line marks *λ*_1se = 7.62699, the most regularized model within one standard error of the minimum deviance.

**Figure 3:**
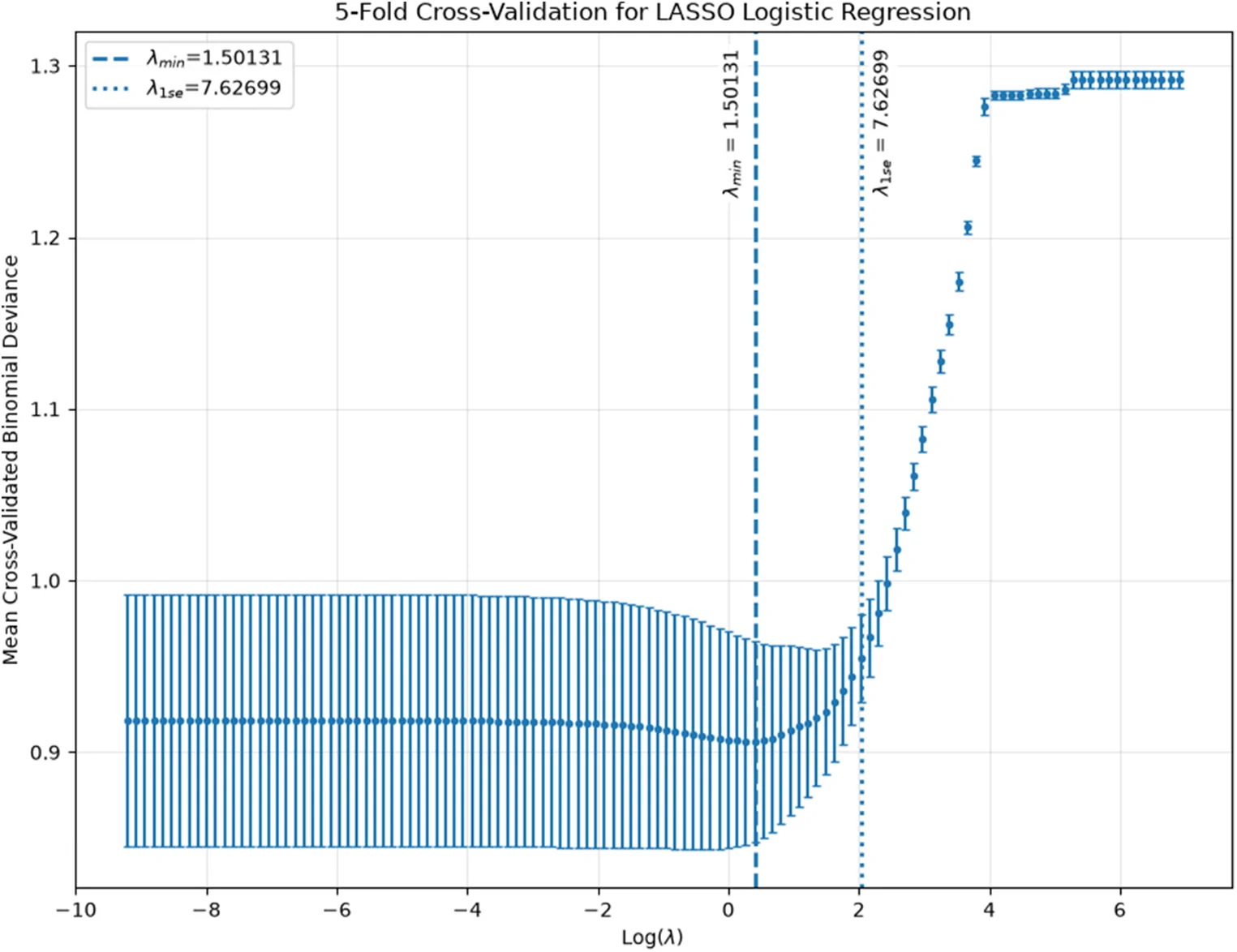
Five-fold cross-validated binomial deviance as a function of log(*λ*). Circles and error bars represent the mean deviance and standard errors across folds, respectively. The vertical dashed and dotted lines mark *λ*_min = 1.50131 and *λ*_1se = 7.62699, respectively.

Correlation and multicollinearity analyses were further performed for the eight selected features. The correlation heatmap demonstrated generally weak-to-moderate correlations among variables. The strongest correlations were observed between PNI and CRP (r ≈ −0.47) and between PNI and MAP (r ≈ 0.30), whereas the absolute correlation coefficients among the remaining variables were relatively low. Variance inflation factor (VIF) analysis showed that all eight variables had VIF values substantially below 5, with the maximum VIF being approximately 1.58, indicating no significant multicollinearity. The correlation heatmap and VIF analysis are presented in Figures 4, 5.

**Figure 4:**
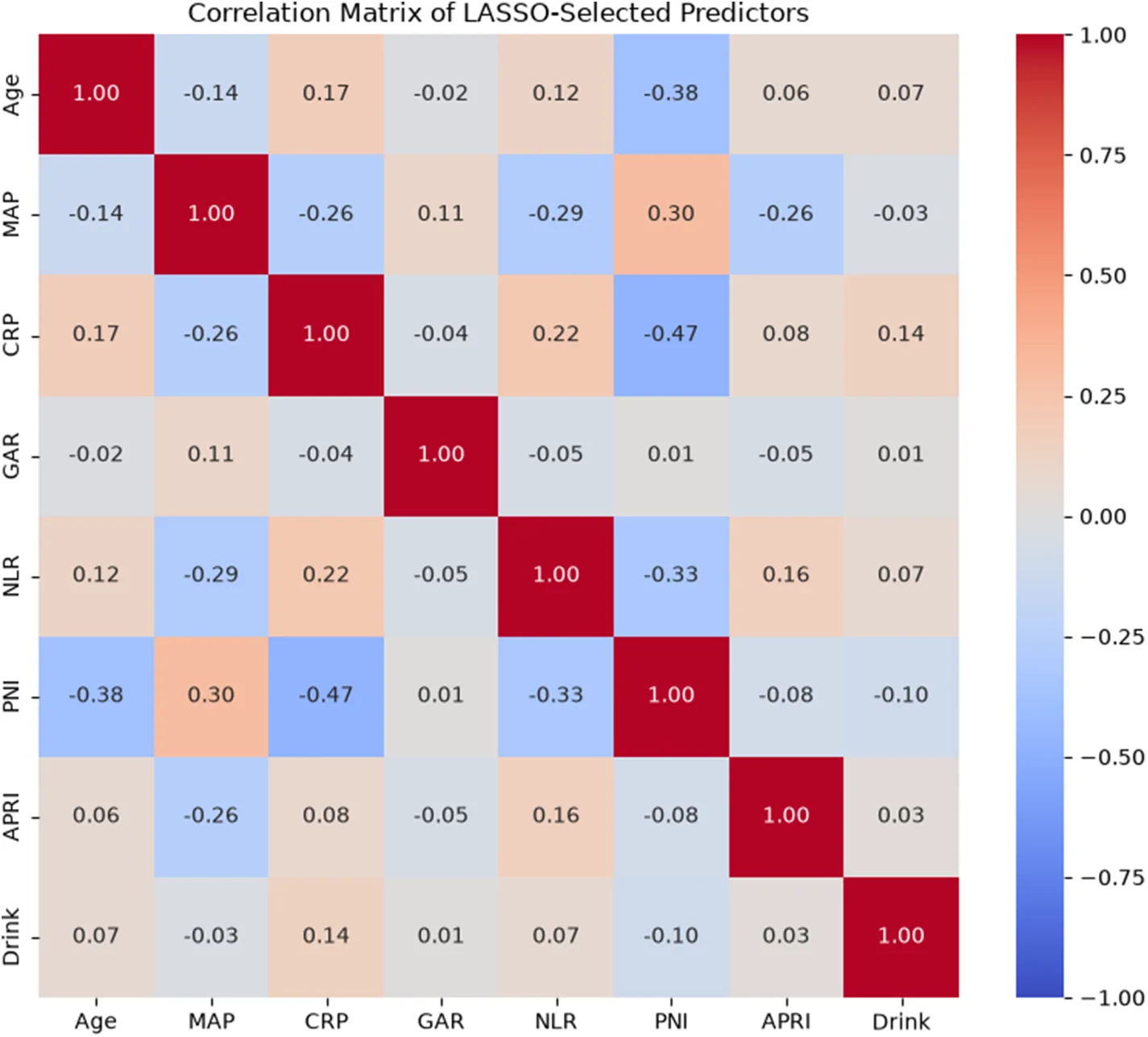
Correlation matrix of LASSO-selected predictors. The heatmap displays the pairwise Pearson correlation coefficients among the eight predictors retained by the LASSO logistic regression model. The color scale ranges from blue (negative correlation) to red (positive correlation), with correlation coefficients annotated in each cell. Predictors include age, mean arterial pressure (MAP), C-reactive protein (CRP), gamma-glutamyl transferase-to-albumin ratio (GAR), neutrophil-to-lymphocyte ratio (NLR), prognostic nutritional index (PNI), aspartate aminotransferase-to-platelet ratio index (APRI), and drink.

**Figure 5:**
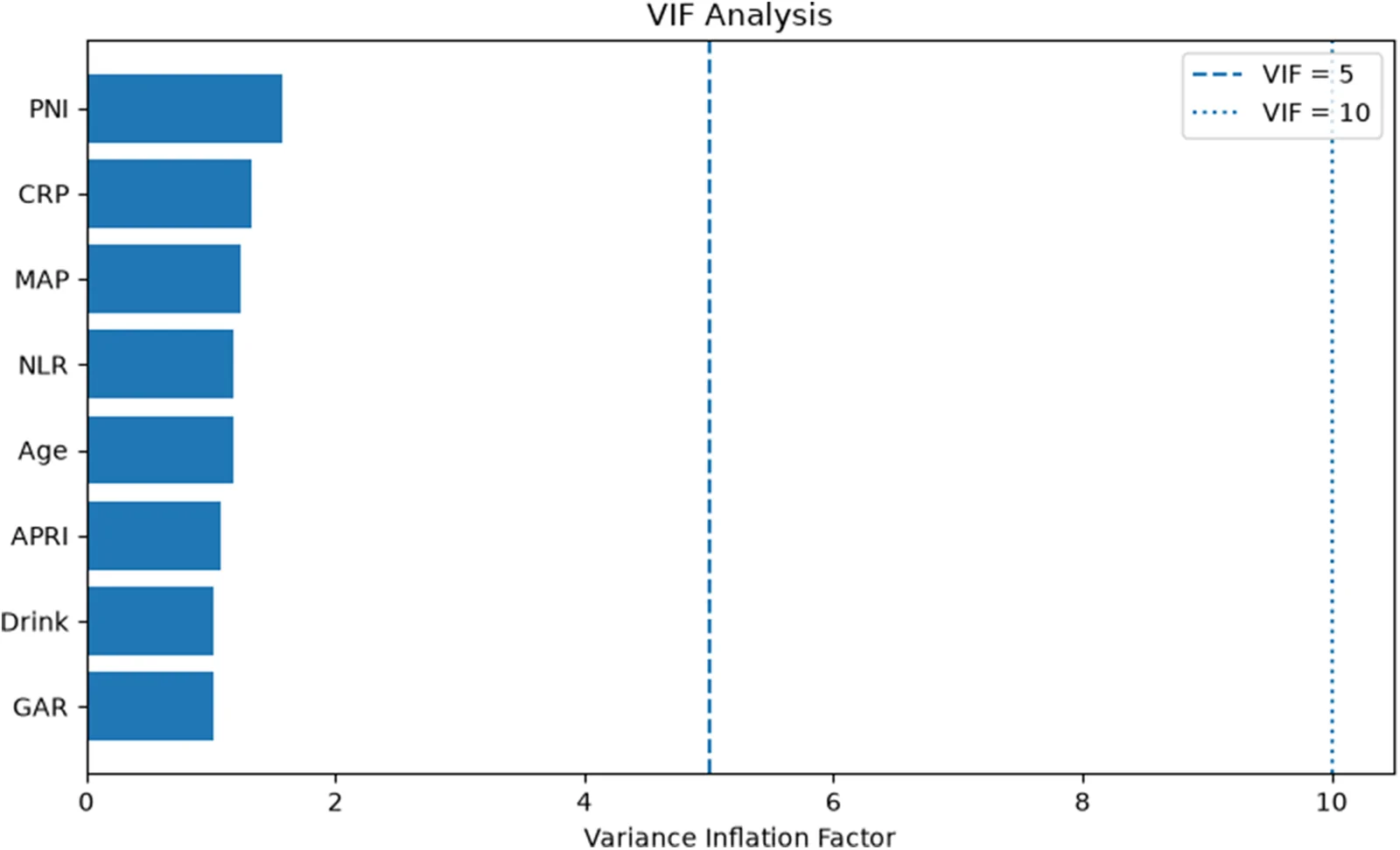
Variance inflation factor (VIF) analysis of LASSO-selected predictors. The bar chart displays the VIF values for the eight predictors retained by the LASSO logistic regression model. The vertical dashed and dotted lines indicate VIF thresholds of 5 and 10, respectively. All VIF values were substantially below 5, indicating no significant multicollinearity among the selected predictors. Predictors include prognostic nutritional index (PNI), C-reactive protein (CRP), mean arterial pressure (MAP), neutrophil-to-lymphocyte ratio (NLR), age, aspartate aminotransferase-to-platelet ratio index (APRI), drink, and gamma-glutamyl transferase-to-albumin ratio (GAR).

### 3.3 Cross-validation performance of five machine learning models in the training cohort

The optimal hyperparameters for RF, SVM, LR, NN, and AdaBoost were determined through five-fold stratified cross-validation combined with grid search, and the detailed parameter settings are summarized in Table 2. The mean cross-validated ROC-AUC values in the training cohort were 0.847 (95% CI, 0.802–0.889) for RF, 0.847 (95% CI, 0.803–0.888) for SVM, 0.842 (95% CI, 0.798–0.883) for LR, 0.828 (95% CI, 0.781–0.869) for NN, and 0.833 (95% CI, 0.789–0.873) for AdaBoost. RF and SVM achieved the highest mean ROC-AUC values (both 0.847). According to the predefined model selection strategy, RF was selected as the final optimal model in cases of equivalent performance and was subsequently used for decision threshold determination, clinical utility assessment, and model interpretation analysis. The training cohort OOF ROC curves for all models are shown in Figure 6.

**Table 2:** Optimized hyperparameters of the five machine learning models.

Model	Optimized hyperparameters	Mean cross-validation ROC-AUC
Random Forest (RF)	n_estimators = 100; max_depth = None; min_samples_split = 5; max_features = sqrt	0.847
Support Vector Machine (SVM)	C = 10; gamma = 0.01; kernel = RBF	0.847
Logistic Regression (LR)	C = 1.0;l1_ratio = 0.0	0.842
Neural Network (NN)	alpha = 0.001; hidden_layer_sizes = (10,); batch_size = 32; learning_rate = constant	0.828
Adaptive boosting (AdaBoost)	n_estimators = 200; learning_rate = 1	0.833

ROC-AUC, area under the receiver operating characteristic curve. Hyperparameters were optimized using five-fold stratified cross-validation combined with grid search in the training cohort, with ROC-AUC used as the optimization criterion. The reported mean cross-validation ROC-AUC values represent the average performance across five validation folds.

**Figure 6:**
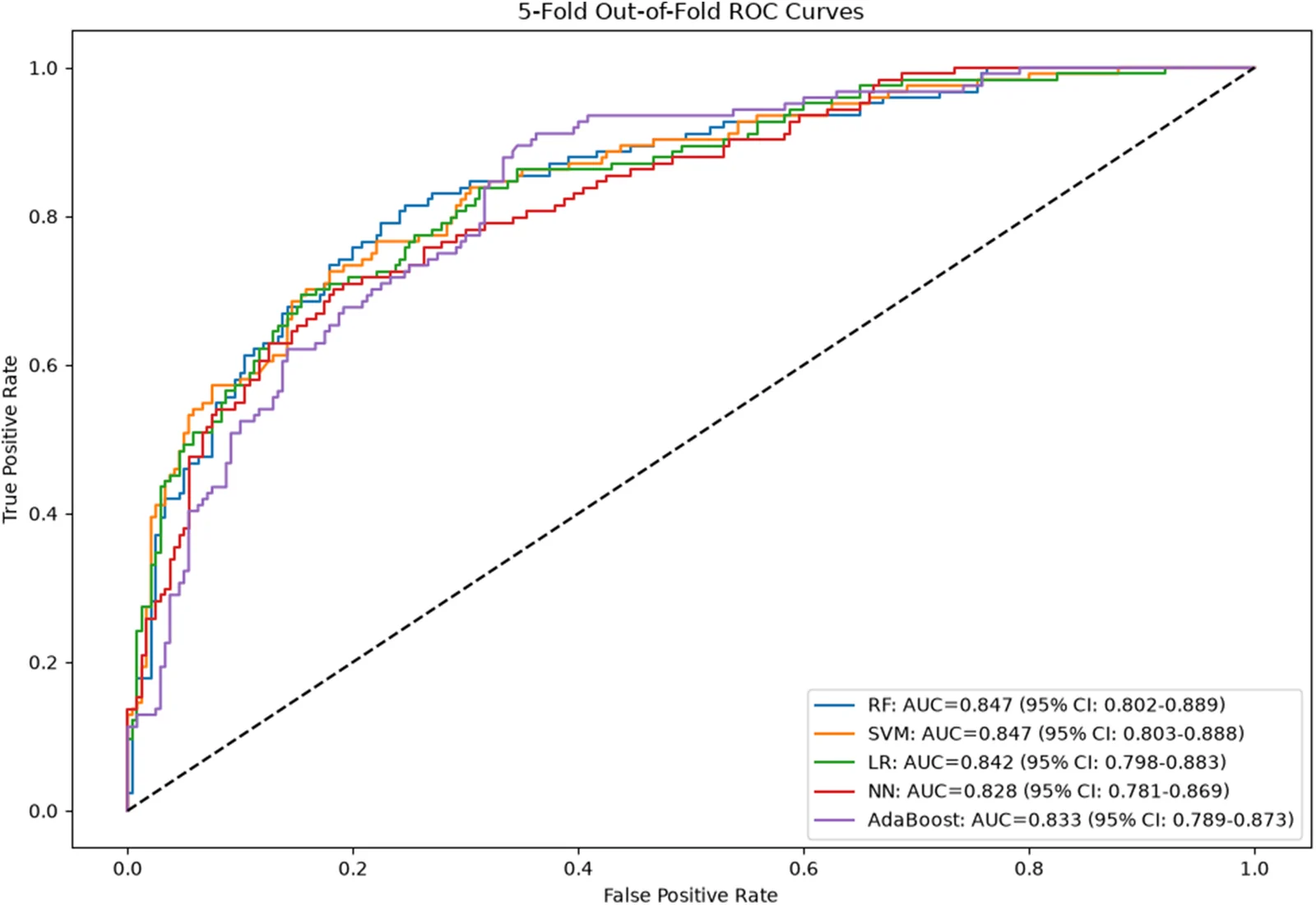
Comparison of receiver operating characteristic (ROC) curves for multiple models on the training Set. The Area Under the Curve (AUC) with its 95% confidence interval (CI) is reported for each model. RF, random forest; SVM, support vector machine; LR, logistic regression; NN, neural network; AdaBoost, adaptive boosting.

Based on RF OOF predicted probabilities in the training cohort, the Youden index was calculated to determine the optimal decision threshold. The selected threshold was 0.342, corresponding to an OOF sensitivity of 0.815 and specificity of 0.754. This threshold was fixed and applied to subsequent independent test-set evaluation without further optimization using test-set information.

### 3.4 Discriminative performance of the models in the independent test set

In the independent test cohort, all five machine learning models demonstrated good discriminative performance. The ROC-AUC values of RF, SVM, LR, NN, and AdaBoost were 0.820 (95% CI, 0.720–0.906), 0.795 (95% CI, 0.687–0.890), 0.798 (95% CI, 0.697–0.887), 0.797 (95% CI, 0.690–0.884), and 0.831 (95% CI, 0.738–0.913), respectively. The ROC curves of the five models in the test cohort are shown in Figure 7.

**Figure 7:**
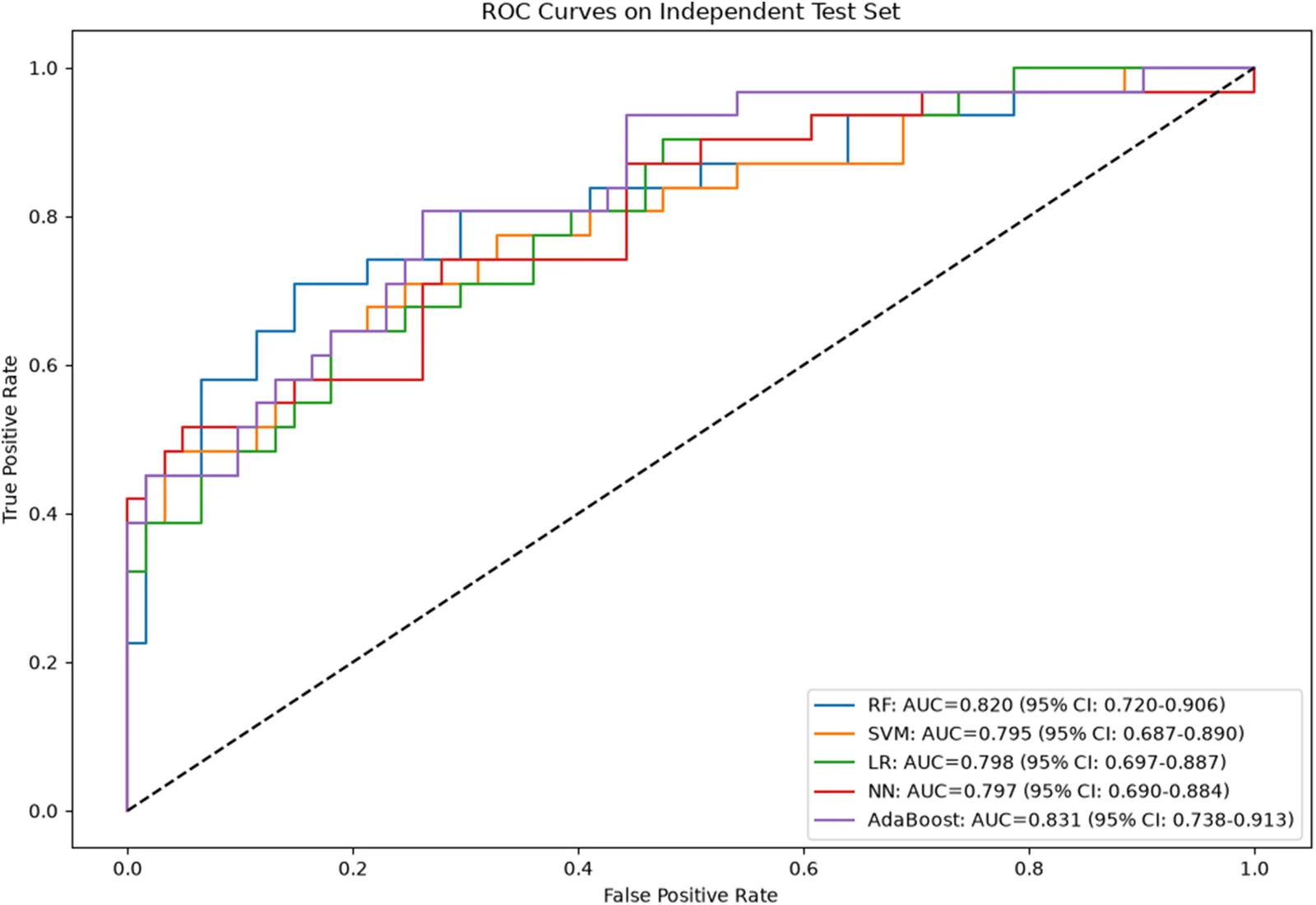
Comparison of receiver operating characteristic (ROC) curves for multiple models on the test Set. The Area Under the Curve (AUC) with its 95% confidence interval (CI) is reported for each model. RF, random forest; SVM, support vector machine; LR, logistic regression; NN, neural network; AdaBoost, adaptive boosting.

Using the fixed classification threshold of 0.342 derived from the training-set OOF predictions, the RF model achieved an accuracy of 72.8%, sensitivity of 80.6%, specificity of 68.9%, PPV of 56.8%, NPV of 87.5%, and an F1-score of 0.667 in the independent test cohort. The corresponding confusion matrix, shown in Figure 8, comprised 25 true-positive, 42 true-negative, 19 false-positive, and 6 false-negative cases. At this prespecified threshold, the RF model showed relatively high sensitivity for identifying patients who developed new-onset AOI within 48 h after admission, while maintaining moderate specificity for patients who did not develop AOI.

**Figure 8:**
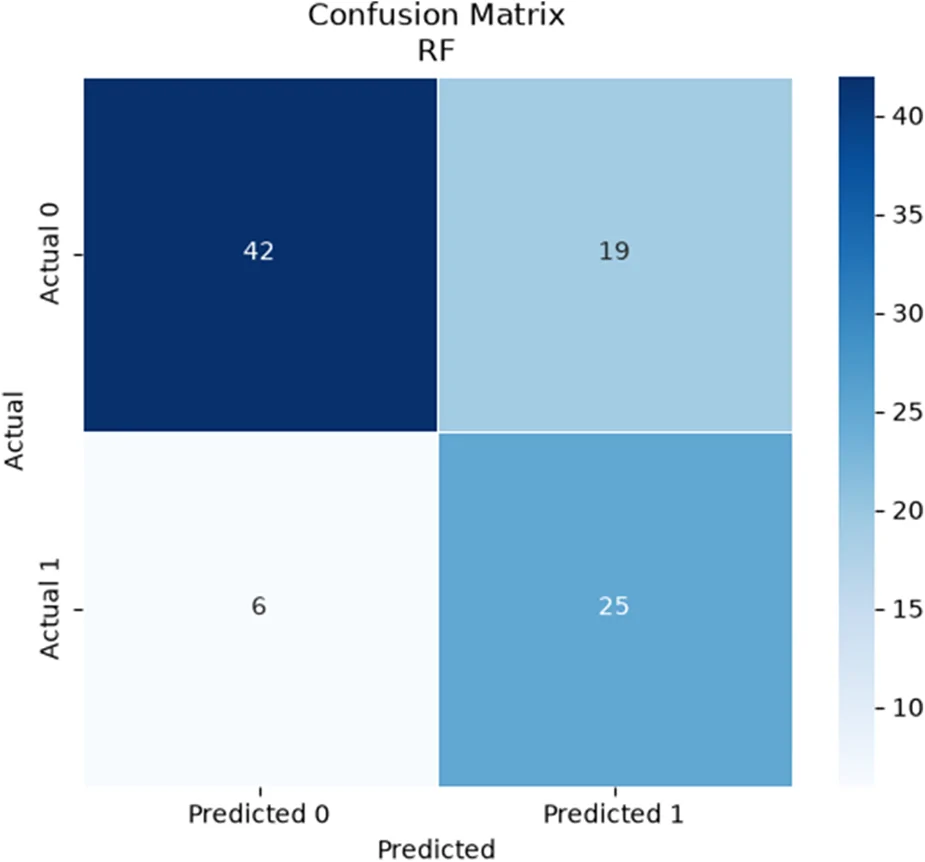
Confusion matrix for the optimal random forest (RF) model on the test set. The matrix compares the actual outcomes (rows) with the model-predicted outcomes (columns). Class ‘1’ indicates the presence of acute organ injury, and Class ‘0’ indicates its absence. Each cell displays the number of cases corresponding to each combination of actual and predicted classes.

### 3.5 Precision-recall analysis, calibration performance, threshold sensitivity analysis, and decision curve analysis

In the independent test cohort, the AP values were 0.769, 0.751, 0.732, 0.757, and 0.771 for RF, SVM, LR, NN, and AdaBoost, respectively. The precision-recall curves of all five models remained above the baseline prevalence of AOI in the test cohort (33.7%), indicating that all models achieved meaningful discrimination for predicting new-onset AOI within 48 h. RF and AdaBoost demonstrated relatively better performance in terms of AP. The precision-recall curves are presented in Figure 9.

**Figure 9:**
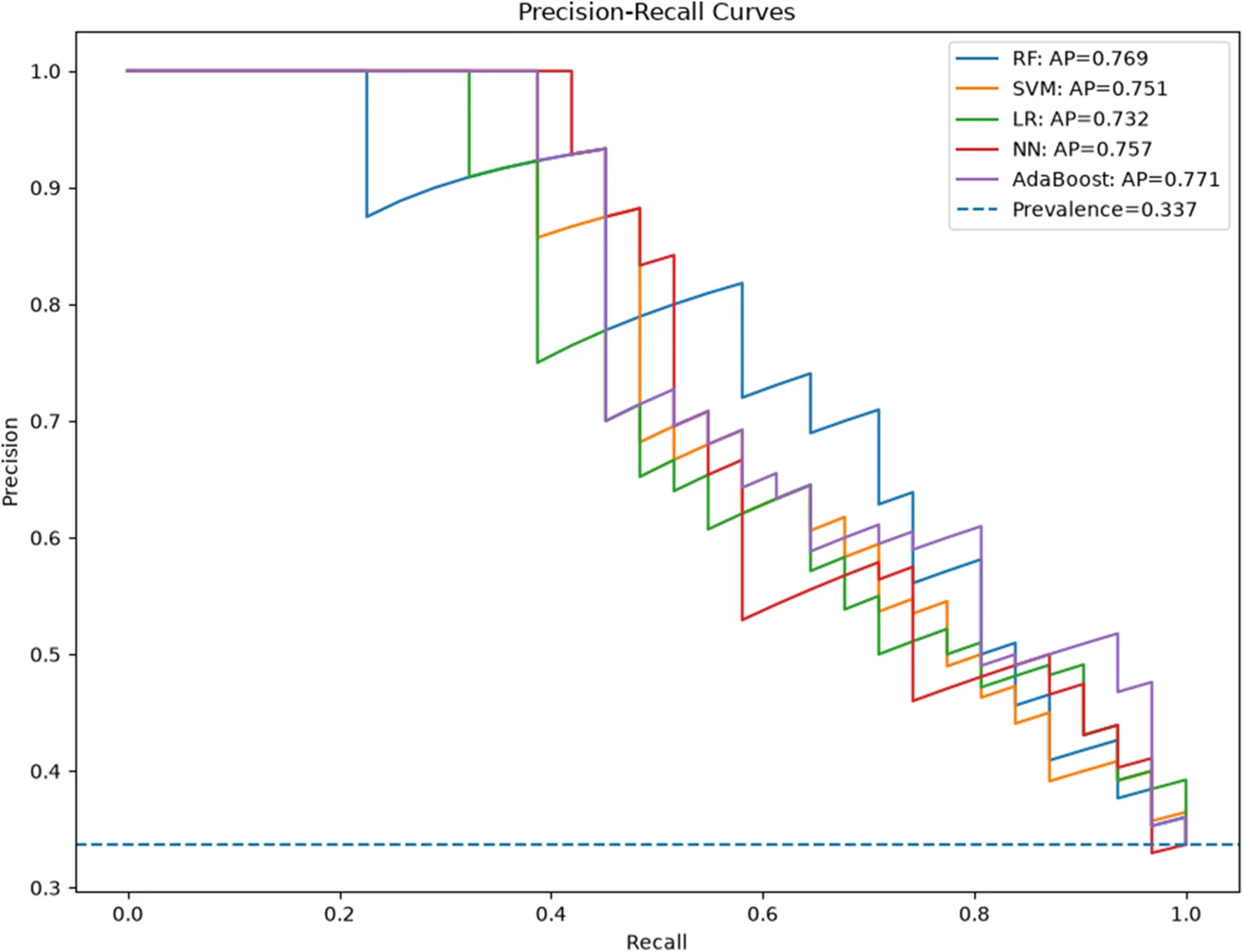
Precision-Recall (PR) curves of the predictive models. The precision-recall plot demonstrates the trade-off between precision (*y*-axis) and recall (*x*-axis) across different probability thresholds for each model. The Average Precision (AP) score, which summarizes the overall performance across all thresholds (with higher values indicating better performance), is annotated for each model in the legend. RF, random forest; SVM, support vector machine; LR, logistic regression; NN, neural network; AdaBoost, adaptive boosting.

Regarding calibration performance, RF achieved the lowest Brier score (0.156), followed by SVM (0.159), LR (0.166), NN (0.169), and AdaBoost (0.215). The Hosmer–Lemeshow goodness-of-fit test for RF showed no significant deviation from good calibration (*p* = 0.711). Among the five models, RF, SVM, and LR did not demonstrate significant calibration misfit, whereas NN and AdaBoost showed relatively poorer calibration performance. For RF, the calibration intercept was 0.014 (95% CI, −0.554 to 0.582), and the calibration slope was 1.252 (95% CI, 0.651–1.853). Overall, RF demonstrated relatively favorable agreement between predicted probabilities and observed outcomes. The calibration curves are shown in Figure 10.

**Figure 10:**
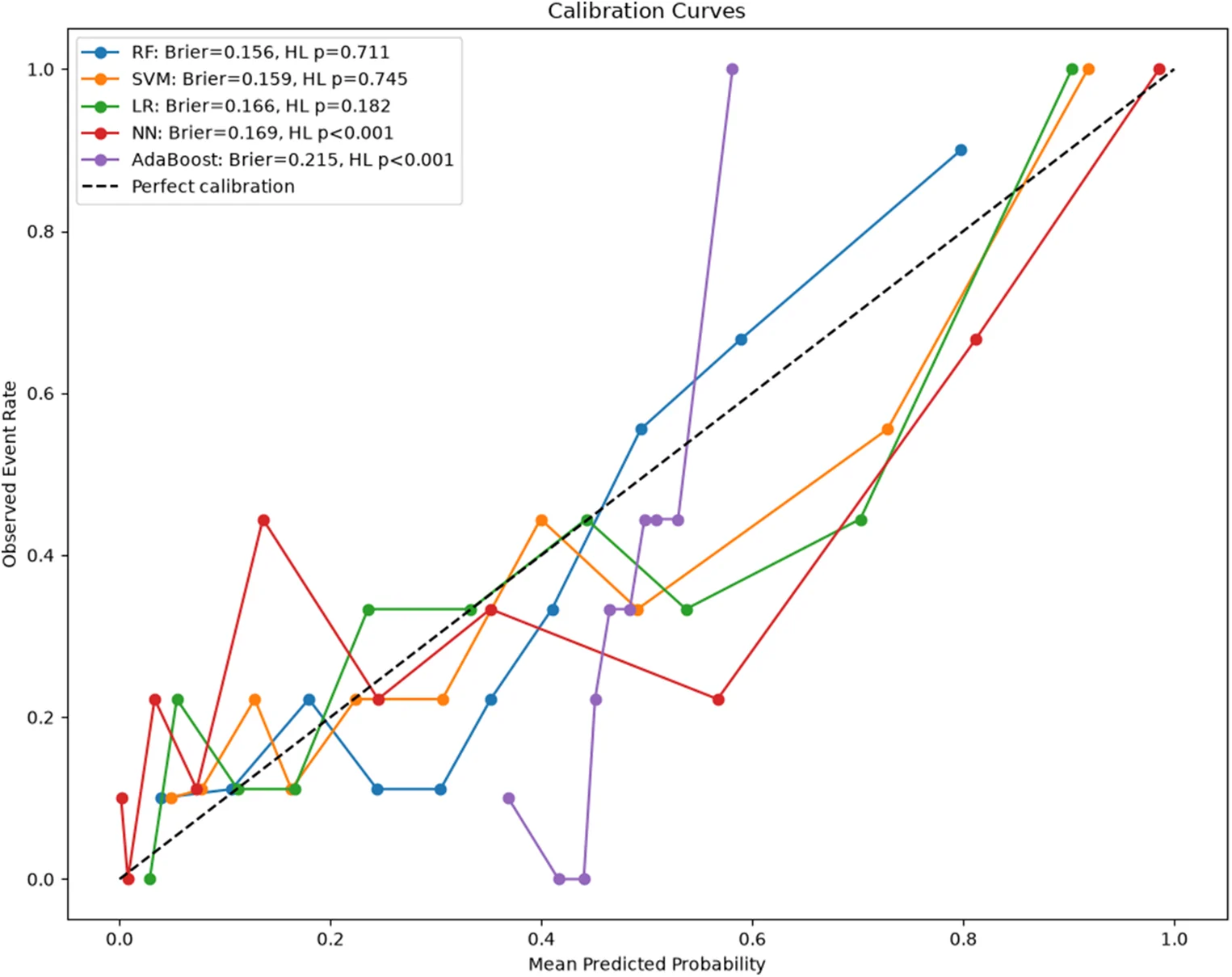
Calibration curves for the five predictive models. The plot illustrates the relationship between the mean predicted probability (*x*-axis) and the observed fraction of positive outcomes (*y*-axis). The dashed 45° line represents ideal calibration; closer proximity of a model's curve to this line indicates superior calibration performance. The Brier score and the Hosmer-Lemeshow test *p* value for each model are annotated in the figure. The Brier score measures the overall calibration error, with lower values indicating better calibration. RF, random forest; SVM, support vector machine; LR, logistic regression; NN, neural network; AdaBoost, adaptive boosting.

Threshold sensitivity analysis of RF demonstrated that, with increasing decision thresholds from 0.05 to 0.95, sensitivity generally decreased, whereas specificity and PPV showed increasing trends. NPV gradually declined with increasing thresholds, while accuracy remained relatively higher within the intermediate threshold range. The F1-score reached relatively higher values at thresholds approximately between 0.45 and 0.55. The OOF-derived Youden threshold of 0.342 was located within a region showing a balanced trade-off between sensitivity and specificity. The results of threshold sensitivity analysis are presented in Figure 11.

**Figure 11:**
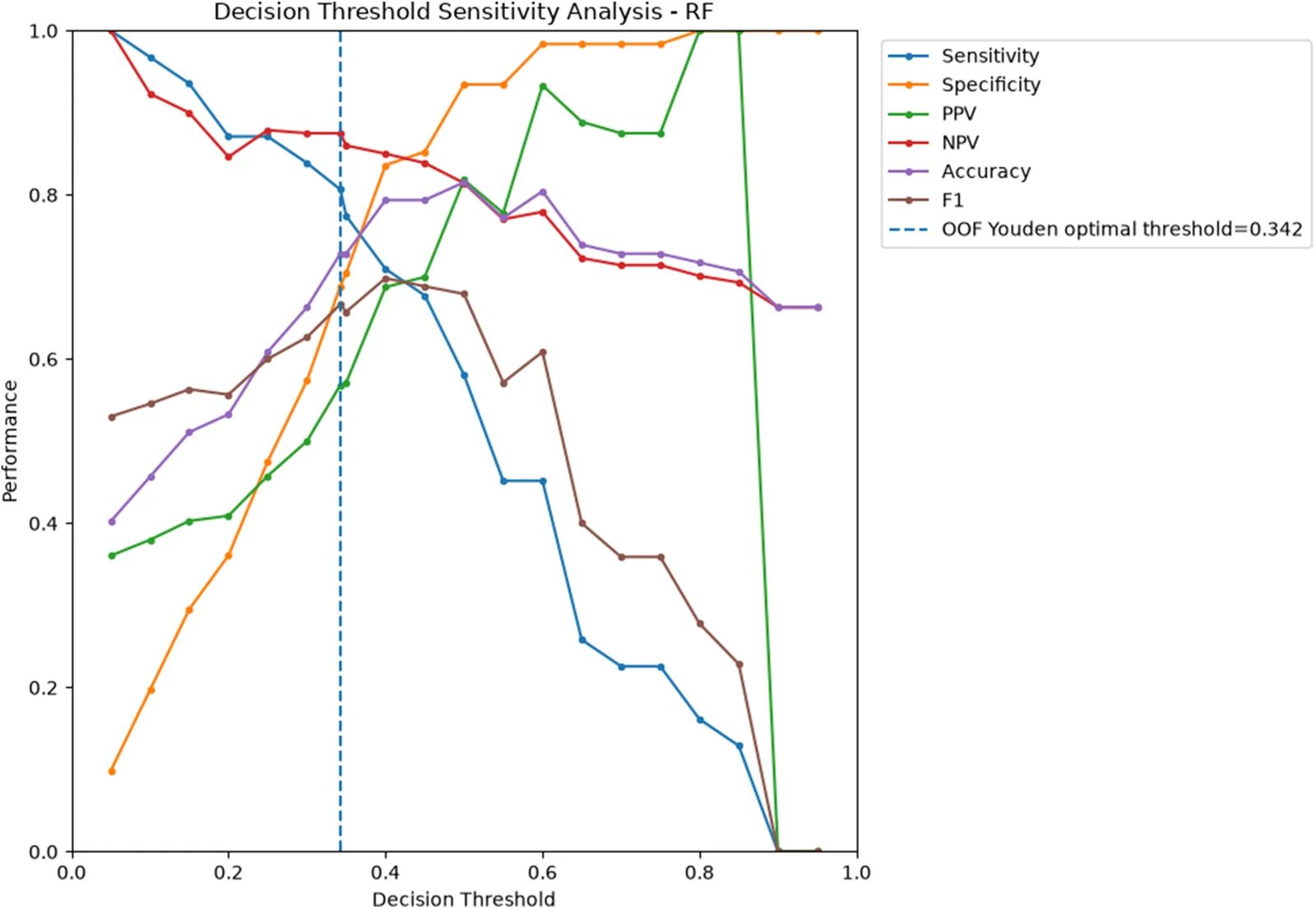
Decision threshold sensitivity analysis for the optimal random forest (RF) model on the test set. The plot illustrates the performance of the RF model across a range of decision thresholds (*x*-axis), with corresponding metrics (*y*-axis) including sensitivity, specificity, positive predictive value (PPV), negative predictive value (NPV), accuracy, and F1-score. The vertical dashed line indicates the Youden optimal threshold of 0.342, which was determined from the five-fold out-of-fold predictions in the training set and applied to the test set.

Decision curve analysis demonstrated that RF maintained a positive net benefit across the evaluated threshold probability range of 0.01–0.80 and generally outperformed the “treat-none” strategy across most threshold probabilities. When the threshold probability exceeded approximately 0.34, the net benefit of the “treat-all” strategy declined below zero, whereas RF continued to provide positive net benefit. Therefore, within this range, RF showed greater potential clinical utility compared with the two extreme treatment strategies. The OOF-derived Youden threshold of 0.342 was located near an important transition region of the DCA curve. At this threshold probability, RF achieved a clinical net benefit of approximately 0.164, with a corresponding 95% CI of 0.044–0.275 estimated using 1,000 bootstrap resampling iterations. These findings suggest that at the optimal decision threshold, RF could provide meaningful clinical benefit and maintain robustness in decision-making. The detailed net benefit estimates and 95% CI are presented in Figure 12.

**Figure 12:**
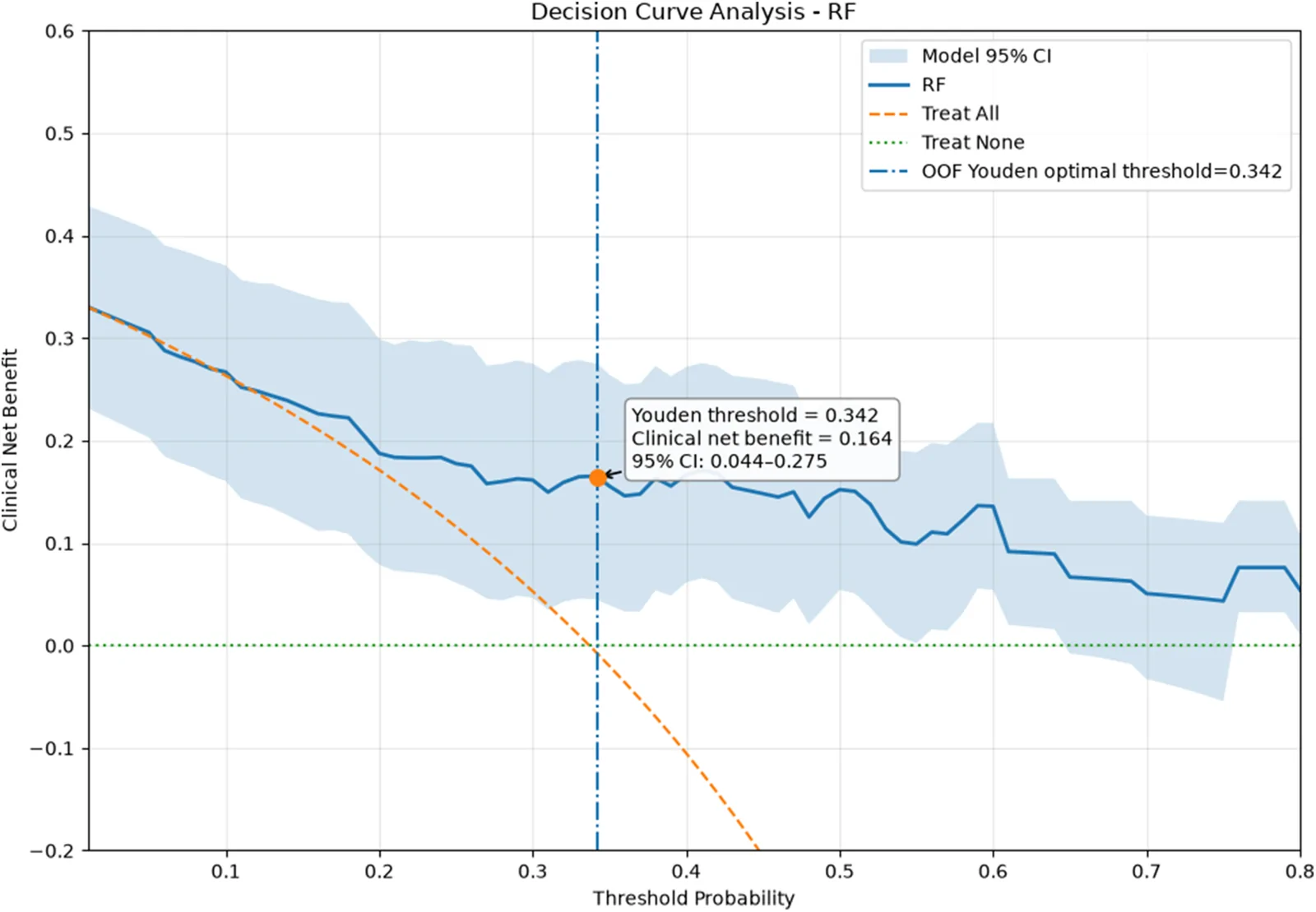
Decision curve analysis (DCA) for the optimal random forest (RF) model on the test set. The plot displays the net clinical benefit (*y*-axis) of the RF model across a range of threshold probabilities (*x*-axis), with the shaded area representing the 95% bootstrap confidence interval. The blue solid line represents the RF model; the dashed line represents the “treat all” strategy; and the dotted line represents the “treat none” strategy. The vertical dashed line marks the Youden optimal threshold of 0.342 derived from training-set out-of-fold predictions.

### 3.6 SHAP-based interpretation of the RF model

Global SHAP analysis of the RF model demonstrated that the eight final predictive features were ranked according to their mean absolute SHAP values as follows: CRP, APRI, MAP, PNI, GAR, Age, NLR, and Drink (Figure 13A). CRP and APRI showed the highest mean absolute SHAP values, indicating the greatest overall contribution to model output, followed by MAP, PNI, GAR, Age, NLR, and Drink.

**Figure 13:**
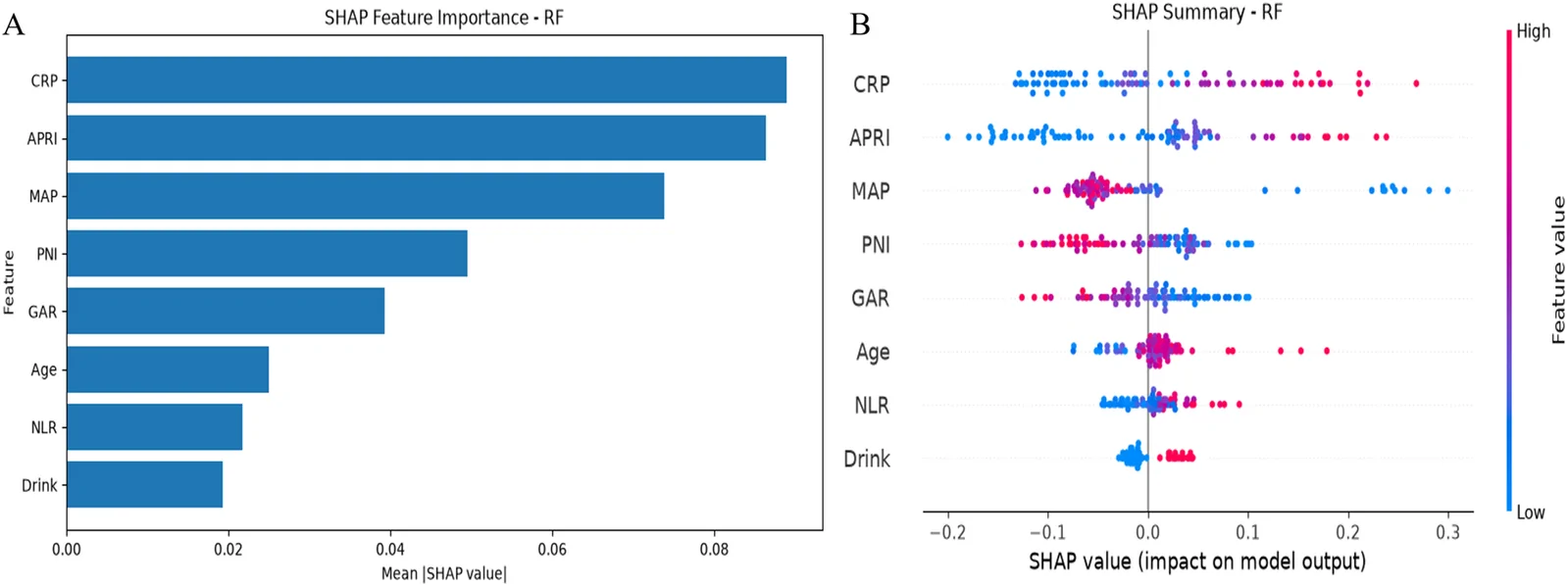
Interpretation of the optimal random forest (RF) model using SHapley additive exPlanations (SHAP). **(A)** Bar plot of mean SHAP value importance for the top features. The features are ranked in descending order of their mean absolute SHAP values. **(B)** Beeswarm plot of SHAP values for the top features. The plot summarizes the distribution of feature values across all samples and their impact on the model output. Each point represents an individual sample, with color indicating the normalized feature value (red: high value, associated with higher risk; blue: low value, associated with lower risk). CRP, C-reactive protein; APRI, aspartate aminotransferase-to-platelet ratio index; MAP, mean arterial pressure; PNI, prognostic nutritional index; GAR, gamma-glutamyl transferase-to-albumin ratio; NLR, neutrophil-to-lymphocyte ratio.

The SHAP beeswarm plot further illustrated the association between feature values and model output. Higher CRP and APRI values were predominantly distributed in the positive SHAP region, indicating a tendency toward increased model prediction probability. Lower MAP values were more frequently associated with positive SHAP contributions, whereas higher MAP values were mainly distributed in the negative SHAP region. PNI, Age, NLR, and GAR also demonstrated distinct contribution patterns, with lower PNI, higher Age, and higher NLR values more frequently located in the positive SHAP region, whereas lower GAR values were mainly associated with positive SHAP contributions. The overall SHAP contribution of Drink showed a predominantly positive direction (Figure 13B). These findings indicate that different feature values exerted heterogeneous directional effects on model predictions.

Individual SHAP analysis was further performed to illustrate the prediction process in representative true-positive and true-negative cases. In the true-positive case, the final predicted probability was 0.86, exceeding the predefined Youden threshold of 0.342. SHAP analysis demonstrated that multiple features jointly contributed to the final prediction output, with CRP, MAP, PNI, NLR, APRI, and Age representing major contributors toward increasing the model prediction probability, whereas other variables showed opposing contributions. The cumulative effect of multiple feature contributions resulted in a predicted probability above the classification threshold, leading to a positive prediction (Figure 14A).

**Figure 14:**
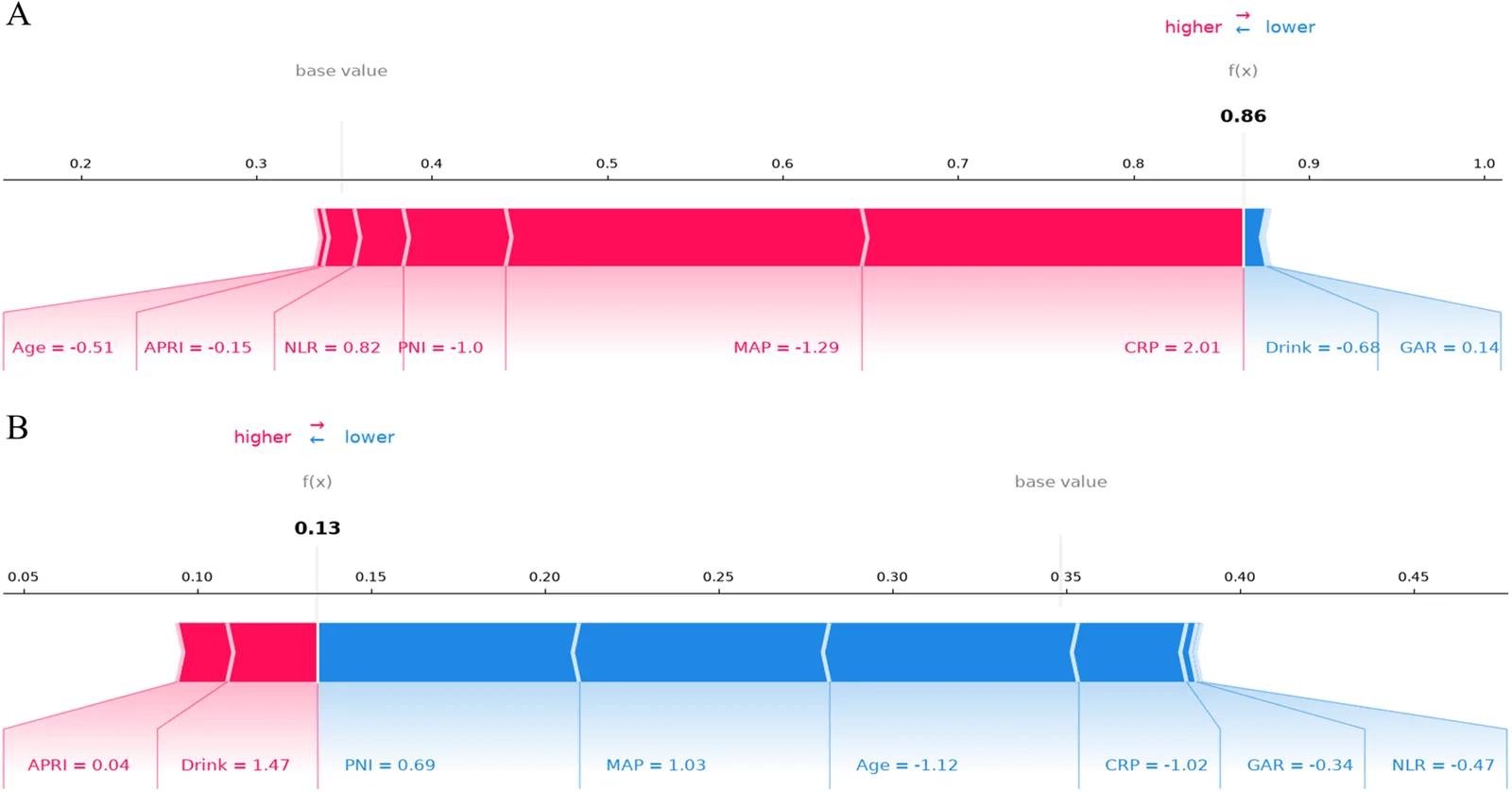
Force plots of individual predictions from the random forest (RF) model for acute organ injury. Force plots illustrate how each feature contributes to pushing the model's base value (the average prediction over the dataset) towards the final prediction for an individual sample. Features pushing the prediction higher are shown in red, while those pushing it lower are shown in blue. The length of each bar corresponds to the magnitude of the feature's contribution. f**(x)** represents the final predicted probability for the specific sample shown. **(A)** Explanation for a representative true-positive (TP) sample. The plot demonstrates how the combined effect of key features pushes the model's output well above the decision threshold, leading to a high-risk prediction. **(B)** Explanation for a representative true-negative (TN) sample. The plot demonstrates how the combined effect of key features pushes the model's output well below the decision threshold, leading to a low-risk prediction.

In the true-negative case, the final predicted probability was 0.13, below the predefined threshold. Compared with the true-positive case, the pattern of Shapley contributions differed substantially. PNI, MAP, Age, CRP, GAR, and NLR showed predominantly negative contributions, whereas APRI and Drink contributed positively to the model output. The combined effect of multiple feature contributions resulted in a lower model prediction probability and a negative classification (Figure 14B).

## 4 Discussion

This study developed and internally validated five machine learning models for predicting incident AOI within 48 h after hospital admission in patients with CAAC. The RF model was selected according to prespecified cross-validation criteria, while differences in test-set performance among the models were small and were not formally compared statistically. By interpreting this “black-box” model using the SHAP framework, we not only identified the most influential predictive variables—including CRP, APRI, MAP, PNI, GAR, Age, NLR, and Drink—but also quantified their contributions to the model output, thereby enhancing the clinical interpretability of the results.

The incidence of new-onset AOI within 48 h after admission in our CAAC cohort was 34.0%. Direct comparison with previously reported rates should be interpreted cautiously because differences in study populations, outcome definitions, assessment time points, and study designs may affect the reported incidence. In addition, regional epidemiological characteristics, healthcare accessibility, diagnostic workflows, and potential selection bias inherent in retrospective studies may also contribute to variations across studies. Therefore, the incidence observed in our cohort should be interpreted within the context of the predefined study population and observation period.

CRP was identified as one of the most influential predictors in our machine-learning model, highlighting the important role of systemic inflammation in the development of AOI among patients with CAAC. As a classical acute-phase reactant, CRP is synthesized predominantly by hepatocytes under IL-6 regulation and reflects the intensity of systemic inflammation (23). Elevated CRP is a recognized marker of inflammatory activity in acute cholangitis, and higher levels have been associated with greater disease severity (24, 25). Importantly, persistently elevated CRP may reflect sustained inflammatory activity and an unfavorable clinical course rather than a transient acute-phase response alone (26). Excessive systemic inflammation may promote endothelial barrier disruption and microcirculatory dysfunction, leading to increased vascular permeability, impaired tissue perfusion, and subsequent organ injury (27, 28). Clinical studies in severe infection have linked endothelial glycocalyx injury and impaired microcirculatory perfusion with organ failure, providing biological support for this association (27, 28). These mechanisms may partly explain the strong association between CRP and AOI observed in our model. Therefore, CRP may provide useful information for early risk stratification, while serial CRP assessment may help monitor inflammatory burden and identify patients at risk of clinical deterioration (26).

APRI demonstrated substantial predictive importance in our model, suggesting that hepatic injury and systemic inflammatory disturbances may contribute to the development of AOI in patients with CAAC. APRI, calculated from AST levels relative to the upper limit of normal and platelet counts, was initially developed as a noninvasive marker for assessing hepatic fibrosis in chronic liver disease (29). However, emerging evidence suggests that APRI may also have prognostic and risk-stratification value in acute inflammatory and critical illnesses. In patients with sepsis, elevated APRI has been associated with the development of sepsis-associated liver injury or dysfunction, supporting its potential utility beyond chronic liver disease (30, 31). Biliary obstruction and cholestasis can induce hepatocellular injury and elevations in aminotransferases, including AST, while concurrent infection and systemic inflammation may further aggravate hepatic injury. Meanwhile, thrombocytopenia during severe infection may result from enhanced platelet consumption, coagulation activation, and impaired platelet production, and is closely associated with a dysregulated host response and greater disease severity (32, 33). Therefore, an increased APRI may represent a composite indicator integrating hepatic injury, platelet alterations, and systemic inflammatory burden rather than reflecting isolated liver dysfunction. Hepatic dysfunction during severe infection has been associated with greater organ dysfunction and adverse outcomes (30, 31), which may partly explain the strong predictive contribution of APRI to AOI in our model. Thus, APRI, as an inexpensive and readily available composite biomarker, may provide additional value for early risk stratification of patients with CAAC at risk of AOI.

Our findings are highly consistent with established pathophysiological theories. Reduced MAP is a marker of systemic hypoperfusion, which can directly lead to ischemic injury in organs (particularly the kidneys and intestines) (34), a critical mechanism in sepsis and systemic inflammatory response. Therefore, early aggressive fluid resuscitation and the use of vasoactive agents to maintain adequate MAP could be a core strategy for preventing AOI in CAAC patients (35).

The PNI combines nutritional status (albumin) with immune competence (lymphocyte count). A low PNI signifies a compromised “nutrition-immunity” axis, leaving patients more vulnerable to progression to organ failure due to an inability to mount an effective response to infectious stress (36). This finding underscores the importance of early nutritional assessment and support in CAAC patients.

GAR, a recently proposed composite biomarker, also demonstrated favorable predictive performance in our study. Elevated GGT generally reflects cholestatic and hepatobiliary injury (37), whereas decreased albumin levels may reflect systemic inflammatory burden and diminished physiological reserve during acute illness (38). By integrating these two parameters, GAR may provide a more comprehensive representation of hepatobiliary dysfunction and systemic disease burden. Recent evidence has further demonstrated the independent prognostic value of GAR in hepatobiliary diseases, supporting its potential utility as an integrated biomarker for clinical risk assessment (39). Taken together, our findings suggest that GAR, as an inexpensive and readily available composite indicator, may provide additional value for early risk stratification of CAAC patients at risk of AOI.

Age is a non-modifiable core risk factor. The inherent decline in physiological reserve (physiological senescence), immune dysfunction (immunosenescence), and a higher burden of comorbidities in elderly patients collectively underpin their vulnerability, making them more prone to decompensation upon acute infection (40).

NLR, an established marker reflecting systemic inflammation and immune dysregulation, showed predictive contribution in our model (41). During infection, increased neutrophil counts represent activation of innate immune responses, whereas reduced lymphocyte counts may indicate impaired adaptive immunity or immune suppression (42). Persistent inflammatory activation accompanied by immune dysfunction has been recognized as a critical mechanism contributing to sepsis-associated organ injury (43). Therefore, NLR may serve as an accessible indicator of the inflammatory-immune balance and provide complementary information for early risk assessment of AOI in patients with CAAC.

Drink was also identified as an important predictive factor in our model. Chronic alcohol exposure can promote hepatic injury and disrupt innate and adaptive immune responses, thereby increasing susceptibility to infection-related complications (44). Alcohol-related alterations in the gut microbiota and intestinal barrier may further facilitate microbial translocation and amplify systemic inflammatory responses (45, 46). In patients with CAAC, these alcohol-related hepatic and immune disturbances may reduce physiological resilience to acute infectious stress, thereby increasing vulnerability to disease progression. Therefore, Drink, as an easily obtainable clinical variable, may provide additional information for early risk stratification and individualized management of patients with CAAC.

A major strength and innovation of this study lies in the application of the SHAP framework, which extends the role of machine-learning models from mere prediction toward model interpretation. Although conventional machine-learning algorithms generally exhibit strong predictive performance, their complex and non-transparent structures often limit their clinical applicability. SHAP analysis not only elucidated the direction and magnitude of individual feature contributions to model predictions but also provided insights into potential interactions among variables. For example, the coexistence of heightened inflammatory status (e.g., elevated CRP levels) and impaired circulatory perfusion (e.g., reduced MAP) may synergistically increase the risk of AOI development. This interpretable machine-learning approach provides a theoretical foundation for the future development of individualized risk scoring systems, establishment of dynamic intervention thresholds, and integration of artificial intelligence–assisted decision support into clinical practice.

We fully acknowledge the limitations of this study: 1) Its single-center, retrospective design limits the generalizability of the findings. 2) Although the model demonstrated good internal validation performance, external validation is required to confirm its universal applicability. 3) Retrospective analysis is inevitably subject to missing data for some potentially important variables. In addition, due to the constraints of the retrospective dataset, some variables required by existing scoring systems could not be fully obtained, and future studies should incorporate comparative analyses in this regard. 4) Machine learning models reveal associations, not causation; the mechanisms discussed are largely speculative and require confirmation through fundamental research.

Consequently, future research directions should include: 1) Conducting multi-center prospective studies to validate and refine this predictive model. 2) Integrating the model with hospital information systems to develop a real-time risk tool for clinical decision support. 3) Initiating in-depth basic experimental research to investigate the precise molecular mechanisms by which the identified features contribute to AOI secondary to CAAC, providing clues for novel therapeutic targets.

## 5 Conclusion

In this study, we developed and evaluated machine learning-based predictive models for new-onset AOI within 48 h of admission in patients with CAAC without baseline organ dysfunction. The RF model was selected as the primary model according to prespecified cross-validation criteria. In the test set, differences in predictive performance among the models were small, and no formal pairwise statistical comparisons were performed. The incorporation of SHAP-based interpretability enhanced model transparency by quantifying the contribution of individual predictors, facilitating a more nuanced understanding of the factors associated with early organ injury. These findings support the potential utility of an interpretable machine learning framework for early risk stratification and individualized management in this specific population. Nevertheless, given the single-center and retrospective nature of the current study, prospective multicenter external validation is essential prior to any clinical translation.

## Data Availability

The raw data supporting the conclusions of this article will be made available by the authors, without undue reservation.

## References

[1] Kiriyama S Kozaka K Takada T Strasberg SM Pitt HA Gabata T. Tokyo Guidelines 2018: diagnostic criteria and severity grading of acute cholangitis (with videos). J Hepatobiliary Pancreat Sci. (2018) 25(1):17–30. 10.1002/jhbp.512 29032610

[2] Lavillegrand JR Mercier-Des-Rochettes E Baron E Pène F Contou D Favory R. Acute cholangitis in intensive care units: clinical, biological, microbiological spectrum and risk factors for mortality: a multicenter study. Crit Care. (2021) 25(1):49. 10.1186/s13054-021-03480-1 33549136 PMC7866656

[3] Lee YS Cho KB Park KS Lee JY Lee YJ. Procalcitonin as a decision-supporting marker of urgent biliary decompression in acute cholangitis. Dig Dis Sci. (2018) 63(9):2474–9. 10.1007/s10620-018-4963-1 29480416

[4] Qin YS Li QY Yang FC Zheng SS. Risk factors and incidence of acute pyogenic cholangitis. Hepatobiliary Pancreat Dis Int. (2012) 11(6):650–4. 10.1016/S1499-3872(12)60240-9 23232638

[5] Zhao J Wang B Zhao M Pan X. Clinical and biochemical factors for bacteria in bile among patients with acute cholangitis. Eur J Gastroenterol Hepatol. (2024) 37(1):33–8. 10.1097/MEG.0000000000002849 39292969 PMC11608589

[6] Zhou Y Zhang YQ Huang SJ Liang Y Liang X Wali M. Urgent one-stage endoscopic treatment for choledocholithiasis related moderate to severe acute cholangitis: a propensity score-matched analysis. World J Gastroenterol. (2024) 30(15):2118–27. 10.3748/wjg.v30.i15.2118 38681983 PMC11045478

[7] Sung YK Lee JK Lee KH Lee KT Kang CI. The clinical epidemiology and outcomes of bacteremic biliary tract infections caused by antimicrobial-resistant pathogens. Am J Gastroenterol. (2012) 107(3):473–83. 10.1038/ajg.2011.387 22334249

[8] Zou Q Ding Y Li CS Yang XP. A randomized controlled trial of emergency LCBDE + LC and ERCP + LC in the treatment of choledocholithiasis with acute cholangitis. Wideochir Inne Tech Maloinwazyjne. (2021) 17(1):156–62. 10.5114/wiitm.2021.108214 35251401 PMC8886466

[9] Liu Q Zhou Q Song M Zhao F Yang J Feng X. A nomogram for predicting the risk of sepsis in patients with acute cholangitis. J Int Med Res. (2019) 48(1):300060519866100. 10.1177/0300060519866100 31429338 PMC7140205

[10] Ambale-Venkatesh B Yang X Wu CO Liu K Hundley WG McClelland R. Cardiovascular event prediction by machine learning: the multi-ethnic study of atherosclerosis. Circ Res. (2017) 121(9):1092–101. 10.1161/CIRCRESAHA.117.311312 28794054 PMC5640485

[11] Saux P Bauvin P Raverdy V Teigny J Verkindt H Soumphonphakdy T. Development and validation of an interpretable machine learning-based calculator for predicting 5-year weight trajectories after bariatric surgery: a multinational retrospective cohort SOPHIA study. Lancet Digit Health. (2023) 5(10):e692–702. 10.1016/S2589-7500(23)00135-8 37652841

[12] Zhu J Wang D Liu C Huang R Gao F Feng X. Development and validation of a new prognostic immune-inflammatory-nutritional score for predicting outcomes after curative resection for intrahepatic cholangiocarcinoma: a multicenter study. Front Immunol. (2023) 14:1165510. 10.3389/fimmu.2023.1165510 37063918 PMC10102611

[13] Li H Liu R Li J Li J Wu H Wang G. Prognostic significance of gamma-glutamyl transpeptidase to albumin ratio in patients with intrahepatic cholangiocarcinoma after hepatectomy. J Cell Mol Med. (2022) 26(11):3196–202. 10.1111/jcmm.17321 35481993 PMC9170822

[14] Kidney Disease: Improving Global Outcomes (KDIGO) Acute Kidney Injury Work Group. KDIGO Clinical practice guideline for acute kidney injury. Kidney Int Suppl. (2012) 2(1):1–138. 10.1038/kisup.2012.1

[15] Obadina ET Torrealba JM Kanne JP. Acute pulmonary injury: high-resolution CT and histopathological spectrum. Br J Radiol. (2013) 86(1027):20120614. 10.1259/bjr.20120614 23659926 PMC3922179

[16] Pruinelli L Westra BL Yadav P Hoff A Steinbach M Kumar V. Delay within the 3-hour surviving sepsis campaign guideline on mortality for patients with severe sepsis and septic shock. Crit Care Med. (2018) 46(4):500–5. 10.1097/CCM.0000000000002949 29298189 PMC5851815

[17] Woźnica EA Inglot M Woźnica RK Łysenko L. Liver dysfunction in sepsis. Adv Clin Exp Med. (2018) 27(4):547–51. 10.17219/acem/68363 29558045

[18] Mehta R Chinthapalli K. Glasgow Coma scale explained. Br Med J. (2019) 365:l1296. 10.1136/bmj.l1296 31048343

[19] Thygesen K Alpert JS Jaffe AS Chaitman BR Bax JJ Morrow DA. Fourth universal definition of myocardial infarction (2018). Circulation. 2018 138(20):e618–51. 10.1161/CIR.0000000000000617 30571511

[20] Thygesen K Mair J Giannitsis E Mueller C Lindahl B Blankenberg S. How to use high-sensitivity cardiac troponins in acute cardiac care. Eur Heart J. (2012) 33(18):2252–7. 10.1093/eurheartj/ehs154 22723599

[21] Evans L Rhodes A Alhazzani W Antonelli M Coopersmith CM French C. Surviving sepsis campaign: international guidelines for management of sepsis and septic shock 2021. Intensive Care Med. (2021) 47(11):1181–247. 10.1007/s00134-021-06506-y 34599691 PMC8486643

[22] Singer M Deutschman CS Seymour CW Shankar-Hari M Annane D Bauer M. The third international consensus definitions for sepsis and septic shock (sepsis-3). JAMA. (2016) 315(8):801–10. 10.1001/jama.2016.0287 26903338 PMC4968574

[23] Ngwa DN Pathak A Agrawal A. IL-6 regulates induction of C-reactive protein gene expression by activating STAT3 isoforms. Mol Immunol. (2022) 146:50–6. 10.1016/j.molimm.2022.04.003 35430542 PMC9811655

[24] Lee SH Lee TY Jeong JH Cheon YK. Clinical significance of the neutrophil-lymphocyte ratio as an early predictive marker for adverse outcomes in patients with acute cholangitis. Medicina (Kaunas). (2022) 58(2):255. 10.3390/medicina58020255 35208579 PMC8878991

[25] Ye S Lyu Y Wang B. The predictive value of different laboratory indicators based on the 2018 Tokyo guidelines for the severity of acute cholangitis. J Emerg Med. (2023) 65(4):e280–9. 10.1016/j.jemermed.2023.05.013 37673776

[26] Póvoa P Teixeira-Pinto AM Carneiro AH. C-reactive protein, an early marker of community-acquired sepsis resolution: a multi-center prospective observational study. Crit Care. (2011) 15(4):R169. 10.1186/cc10313 PMC3387609 21762483

[27] Smart L Macdonald SPJ Burrows S Bosio E Arendts G Fatovich DM. Endothelial glycocalyx biomarkers increase in patients with infection during emergency department treatment. J Crit Care. (2017) 42:304–9. 10.1016/j.jcrc.2017.07.001 28822340

[28] Trzeciak S McCoy JV Phillip Dellinger R Arnold RC Rizzuto M Abate NL. Early increases in microcirculatory perfusion during protocol-directed resuscitation are associated with reduced multi-organ failure at 24 h in patients with sepsis. Intensive Care Med. (2008) 34(12):2210–7. 10.1007/s00134-008-1193-6 18594793 PMC2821162

[29] Wai CT Greenson JK Fontana RJ Kalbfleisch JD Marrero JA Conjeevaram HS. A simple noninvasive index can predict both significant fibrosis and cirrhosis in patients with chronic hepatitis C. Hepatology. (2003) 38(2):518–26. 10.1053/jhep.2003.50346 12883497

[30] Dou J Zhou Y Cui Y Chen M Wang C Zhang Y. AST-to-Platelet ratio Index as potential early-warning biomarker for sepsis-associated liver injury in children: a database study. Front Pediatr. (2019) 7:331. 10.3389/fped.2019.00331 31497584 PMC6713043

[31] Zhang B Li X Qin Z Dong D Yu W. Initial aspartate aminotransferase-to-platelet ratio Index is associated with sepsis-associated liver dysfunction in adult patients with sepsis: a retrospective cohort study. Indian J Crit Care Med. (2025) 29(11):916–24. 10.5005/jp-journals-10071-25079 41368460 PMC12683549

[32] Bedet A Razazi K Boissier F Surenaud M Hue S Giraudier S. Mechanisms of thrombocytopenia during septic shock: a Multiplex cluster analysis of endogenous sepsis mediators. Shock. (2018) 49(6):641–8. 10.1097/SHK.0000000000001015 29028771

[33] Claushuis TAM Van Vught LA Scicluna BP Wiewel MA Klein Klouwenberg PMC Hoogendijk AJ. Thrombocytopenia is associated with a dysregulated host response in critically ill sepsis patients. Blood. (2016) 127(24):3062–72. 10.1182/blood-2015-11-680744 26956172

[34] Khanna AK Maheshwari K Mao G Liu L Perez-Protto SE Chodavarapu P. Association between mean arterial pressure and acute kidney injury and a composite of myocardial injury and mortality in postoperative critically ill patients: a retrospective cohort analysis. Crit Care Med. (2019) 47(7):910–7. 10.1097/CCM.0000000000003763 30985388

[35] Sacha GL Bauer SR. Optimizing vasopressin use and initiation timing in septic shock: a narrative review. Chest. (2023) 164(5):1216–27. 10.1016/j.chest.2023.07.009 37479058 PMC10635838

[36] Liu CC Liu PH Chen HT Chen JY Lee CW Cheng WJ. Association of preoperative prognostic nutritional Index with risk of postoperative acute kidney injury: a meta-analysis of observational studies. Nutrients. (2023) 15(13):2929. 10.3390/nu15132929 37447255 PMC10346508

[37] Luo X Lu LG. Progress in the management of patients with cholestatic liver disease: where are we and where are we going? J Clin Transl Hepatol. (2024) 12(6):581–8. 10.14218/JCTH.2023.00519 38974958 PMC11224908

[38] Eckart A Struja T Kutz A Baumgartner A Baumgartner T Zurfluh S. Relationship of nutritional Status, inflammation, and Serum albumin levels during acute illness: a prospective study. Am J Med. (2019) 133(6):713–722.e7. 10.1016/j.amjmed.2019.10.031 31751531

[39] Luo XY Chang KW Ye N Gao CH Zhu QB Liu JP. The predictive value of *γ*-glutamyl transferase to serum albumin ratio in hepatocellular carcinoma patients after liver transplantation. Front Med (Lausanne). (2024) 11:1380750. 10.3389/fmed.2024.1380750 38799149 PMC11122022

[40] Darden DB Moore FA Brakenridge SC Navarro EB Anton SD Leeuwenburgh C. The effect of aging physiology on critical care. Crit Care Clin. (2020) 37(1):135–50. 10.1016/j.ccc.2020.08.006 33190766 PMC8194285

[41] Wu H Cao T Ji T Luo Y Huang J Ma K. Predictive value of the neutrophil-to-lymphocyte ratio in the prognosis and risk of death for adult sepsis patients: a meta-analysis. Front Immunol. (2024) 15:1336456. 10.3389/fimmu.2024.1336456 38562922 PMC10982325

[42] Zahorec R. Neutrophil-to-lymphocyte ratio, past, present and future perspectives. Bratisl Lek Listy. (2021) 122(7):474–88. 10.4149/BLL_2021_078 34161115

[43] Van Der Poll T Shankar-Hari M Wiersinga WJ. The immunology of sepsis. Immunity. (2021) 54(11):2450–64. 10.1016/j.immuni.2021.10.012 34758337

[44] Crotty K Anton P Coleman LG Morris NL Lewis SA Samuelson DR. A critical review of recent knowledge of alcohol's Effects on the immunological response in different tissues. Alcohol Clin Exp Res (Hoboken). (2022) 47(1):36–44. 10.1111/acer.14979 36446606 PMC9974783

[45] Chen L Zhu Y Hou X Yang L Chu H. The role of gut Bacteria and fungi in alcohol-associated liver disease. Front Med (Lausanne). (2022) 9:840752. 10.3389/fmed.2022.840752 35308525 PMC8927088

[46] Maccioni L Gao B Leclercq S Pirlot B Horsmans Y De Timary P. Intestinal permeability, microbial translocation, changes in duodenal and fecal microbiota, and their associations with alcoholic liver disease progression in humans. Gut Microbes. (2020) 12(1):1782157. 10.1080/19490976.2020.1782157 32588725 PMC7524402

